# Uncoupling sodium channel dimers restores the phenotype of a pain‐linked Na_v_1.7 channel mutation

**DOI:** 10.1111/bph.15196

**Published:** 2020-08-24

**Authors:** Annika H. Rühlmann, Jannis Körner, Ralf Hausmann, Nikolay Bebrivenski, Christian Neuhof, Silvia Detro‐Dassen, Petra Hautvast, Carène A. Benasolo, Jannis Meents, Jan‐Philipp Machtens, Günther Schmalzing, Angelika Lampert

**Affiliations:** ^1^ Institute of Physiology Uniklinik RWTH Aachen University Pauwelsstrasse 30 Aachen Deutschland 52074 Germany; ^2^ Department of Anaesthesiology, Medical Faculty Uniklinik RWTH Aachen University Pauwelsstrasse 30 Aachen Deutschland 52074 Germany; ^3^ Institute of Clinical Pharmacology Uniklinik RWTH Aachen University Pauwelsstrasse 30 Aachen Deutschland 52074 Germany; ^4^ Forschungszentrum Jülich Institute of Biological Information Processing (IBI‐1), Molekular‐ und Zellphysiologie, and JARA‐HPC Jülich Germany

**Keywords:** biophysics, channel gating, patch‐clamp, molecular simulations, mutagenesis

## Abstract

**Background and Purpose:**

The voltage‐gated sodium channel Na_v_1.7 is essential for adequate perception of painful stimuli. Mutations in the encoding gene, *SCN9A*, cause various pain syndromes in humans. The hNa_v_1.7/A1632E channel mutant causes symptoms of erythromelalgia and paroxysmal extreme pain disorder (PEPD), and its main gating change is a strongly enhanced persistent current. On the basis of recently published 3D structures of voltage‐gated sodium channels, we investigated how the inactivation particle binds to the channel, how this mechanism is altered by the hNa_v_1.7/A1632E mutation, and how dimerization modifies function of the pain‐linked mutation.

**Experimental Approach:**

We applied atomistic molecular simulations to demonstrate the effect of the mutation on channel fast inactivation. Native PAGE was used to demonstrate channel dimerization, and electrophysiological measurements in HEK cells and *Xenopus laevis* oocytes were used to analyze the links between functional channel dimerization and impairment of fast inactivation by the hNa_v_1.7/A1632E mutation.

**Key Results:**

Enhanced persistent current through hNa_v_1.7/A1632E channels was caused by impaired binding of the inactivation particle, which inhibits proper functioning of the recently proposed allosteric fast inactivation mechanism. hNa_v_1.7 channels form dimers and the disease‐associated persistent current through hNa_v_1.7/A1632E channels depends on their functional dimerization status: Expression of the synthetic peptide difopein, a 14‐3‐3 inhibitor known to functionally uncouple dimers, decreased hNa_v_1.7/A1632E channel‐induced persistent currents.

**Conclusion and Implications:**

Functional uncoupling of mutant hNa_v_1.7/A1632E channel dimers restored their defective allosteric fast inactivation mechanism. Our findings support the concept of sodium channel dimerization and reveal its potential relevance for human pain syndromes.

**Abbreviations**
CIP: congenital insensitivity to pain
DDM: *n*‐dodecyl β‐d‐maltoside
DI–DIV: domain I–IV
Difopein: dimeric‐fourteen‐three‐three‐peptide inhibitor
EM: electron microscopy
hrCN: high resolution clear native
LiDS: lithium dodecyl sulfate
NG310: lauryl maltose neopentyl glycol
PAGE: Polyacrylamide gel electrophoresis
PDB: protein data bank
PEPD: paroxysmal extreme pain disorder
S1–S6: segment 1–6
TEVC: two‐electrode *voltage‐clamp*
Trp: transient receptor potential cation channels
VGSC: voltage‐gated sodium channels
VSD: voltage‐sensing domain
WT: wild type

**What is already known**

- Mutations in sodium channels leading to chronic pain syndromes often affect their fast inactivation.
- Fast inactivation is mediated by the inactivation particle of the sodium channel protein.

**What does this study add**

- We provide evidence for a recently proposed allosteric mechanism of fast inactivation.
- The Na_v_1.7 channel forms dimers, which affects the gating changes, induced by mutation.

**What is the clinical significance**

- Sodium channel dimerization modulates the pain‐linked, mutation‐induced gating changes in Na_v_1.7 channels.
- Detailed molecular knowledge of inactivation mechanisms may improve drug design to modulate Na_v_1.7 channel function.

## 1 INTRODUCTION

Voltage‐gated sodium channels (VGSCs) play a crucial role in the perception of potentially painful signals (Ahern, Payandeh, Bosmans, & Chanda, 2016). Mutations of hNa_v_1.7 channels are linked to pain syndromes, such as inherited erythromelalgia (IEM) or paroxysmal extreme pain disorder (PEPD) (Lampert, O'Reilly, Reeh, & Leffler, 2010). Patients with complete loss‐of‐function mutations of Na_v_1.7 channels show impaired pain perception–congenital insensitivity to pain (CIP) (Cox et al., 2006; McDermott et al., 2019), stressing the role of these sodium channels in human pain perception.

Human VGSCs consist of four domains (DI to DIV), with six transmembrane segments (S1–S6) (Clairfeuille et al., 2019; Pan et al., 2018; Shen et al., 2017; Shen, Liu, Wu, Lei, & Yan, 2019; Xu et al., 2019; Yan et al., 2017). The central ion pore built by segments S5 and S6 is opened by tethered voltage‐sensing structures formed by segments S1–S4 (voltage‐sensing domain; VSD) (Catterall, 2014; Guy & Seetharamulu, 1986; Payandeh, Scheuer, Zheng, & Catterall, 2011; Sula et al., 2017). VGSCs inactivate within milliseconds of pore opening through a mechanism involving the IFM motif or inactivation particle, comprising three amino acids located in the DIII‐DIV linker (Catterall, 2014).

Until very recently, the IFM motif was thought to cause fast inactivation by binding directly to the cytoplasmic side of the pore and thus blocking ion permeation (Armstrong, Bezanilla, & Rojas, 1973; West et al., 1992), a process described as “hinged lid” mechanism (Eaholtz, Scheuer, & Catterall, 1994; West et al., 1992). However, a new fast inactivation mechanism has been proposed based on the recently published high‐resolution structures of VGSC subtypes Na_v_Pas, Na_v_1.4, and Na_v_1.7: Rather than via direct occlusion, fast inactivation may occur via an allosteric mechanism in which the IFM motif binds to a hydrophobic binding pocket in the periphery of the S6 helical bundle, causing displacement of the DIV S6 helix towards the ion permeation pathway (Clairfeuille et al., 2019; Pan et al., 2018; Shen et al., 2017, 2019; Yan et al., 2017).

Recent studies suggest that the cardiac channel subtype, Na_v_1.5, forms dimers (Clatot et al., 2017, 2018), which are functionally coupled by 14‐3‐3, an abundant intracellular protein (Clatot et al., 2017; Foote & Zhou, 2012; Obsil, Ghirlando, Klein, Ganguly, & Dyda, 2001). The 14‐3‐3 protein contains two binding grooves (Johnson et al., 2010; Obsil & Obsilova, 2011), which are suggested to interact with the linker regions between DI and DII of Na_v_1.5 channels (Clatot et al., 2017). The binding of all known seven human 14‐3‐3 isoforms can be inhibited by the synthetic peptide difopein (**di**meric‐**fo**urteen‐three‐three‐**pe**ptide **in**hibitor) (Masters & Fu, 2001). Difopein inhibition of 14‐3‐3 binding has been suggested to prevent the functional coupling of VGSC dimers (Clatot et al., 2017; Masters & Fu, 2001).

The previously reported gain‐of‐function mutation A1632E of the nociceptive subtype channel, Na_v_1.7, induces a combination of IEM and PEPD in heterozygous carriers and is characterized by incomplete fast inactivation, leading to a prominent persistent current (Eberhardt et al., 2014; Estacion et al., 2008). Several naturally occurring and artificial mutations have been reported at this position, indicating that this region of the VGSC protein regulates the voltage dependence and kinetics of fast inactivation (Eberhardt et al., 2014; Yang et al., 2016). Here, we report our further studies on the hNa_v_1.7/A1632E channels, transfected in HEK cells and *Xenopus laevis* oocytes. Using all‐atom molecular dynamics simulations, we found that increasing the size of the amino acid side chain and adding a negative charge at position 1632 impedes binding of the IFM motif to a hydrophobic binding pocket, thus preventing fast inactivation. This finding supports a newly described allosteric inactivation mechanism suggested by recent cryo‐electron microscopy (cryo‐EM) structures (Pan et al., 2018; Shen et al., 2017, 2019; Yan et al., 2017). Using clear‐native PAGE, we found that hNa_v_1.7 channels form dimers. We present data that reveal how the functional uncoupling of such dimerization significantly reduced the size of the disease‐relevant persistent current in hNa_v_1.7/A1632E channels.

## 2 METHODS

### 2.1 Plasmids

For HEK cells, the pCMV6‐neo‐hNa_v_1.7 vector was previously reported (Klugbauer, Lacinova, Flockerzi, & Hofmann, 1995; Stadler, O'Reilly, & Lampert, 2015). hNa_v_1.7/R896Q and hNa_v_1.7/G375Afs mutations were each introduced into a pCMV6‐neo vector. The pCDNA3‐hNa_v_1.7/A1632E was previously reported (Eberhardt et al., 2014). The pIRES2‐EGFP‐difopein (BioCat, Heidelberg, Germany) and GFP pMax‐GFP (Lonza, Basel, Switzerland) vectors were purchased.

Oocyte expression vectors for cRNA‐encoded proteins were designed using the Vector NTi Deluxe v4.0 software (InforMax). Full‐length hNa_v_1.5 and hNa_v_1.7 coding sequences were each subcloned into the pNKS2 oocyte expression vector (Gloor, Pongs, & Schmalzing, 1995) using the Gateway PCR Cloning System and In‐Fusion HD Cloning respectively. For oocyte expression, difopein cDNA was subcloned from the pIRES2‐EGFP vector into the pNKS2 vector by megaprimer PCR. Human *TRPV1* (transient receptor potential cation channel subfamily V member 1) transcript variant 3 in pDONR201 (DNASU plasmid ID HsCD00081472; corresponding to National Center for Biotechnology Information [NCBI] reference sequence NM_080706.3) was purchased from the DNASU Plasmid Repository (Tempe, AR, USA) and subcloned into the pNKS2 vector by Gateway cloning. hNa_v_1.5, hNa_v_1.7, and hTRPV1 were fused in frame to a C‐terminal GFP coding sequence using megaprimer PCR (Kirsch & Joly, 1998; Perez, Yeam, Jahn, & Kang, 2006) to generate hNa_v_1.5^GFP^, hNa_v_1.7^GFP^, and hTRPV1^GFP^. Codons for a C‐terminal octyl‐histidine (His) tag and a Strep‐tag®II (StrepII; separated by a alanine linker residue from the His‐tag) or Twin‐Strep‐tag® (StrepIII) were inserted according to the QuikChange protocol (Weiner et al., 1994) using Phusion high‐fidelity DNA polymerase and Dpn I restriction endonuclease (New England BioLabs, Frankfurt, Germany) to generate the hNa_v_1.7^His‐StrepIII^ and hNa_v_1.7/A1632E^His‐StrepII^ constructs. For hNa_v_1.7 only, cloning and propagation required the use of stable competent *Escherichia coli* cells (New England Biolabs, Frankfurt, Germany) suitable for plasmids with unstable inserts. Oligonucleotides were purchased from Eurofins Genomics (Ebersberg, Germany). All constructs were verified by restriction pattern analysis and commercial DNA sequencing (Eurofins, Ebersberg, Germany).

### 2.2 Cell culture and transfection

Two HEK cell lines were used: HEK293T cells (HEK293T, RRID: CVCL_0063) and a HEK cell line stably expressing hNa_v_1.7/WT (from now on referred to as Na_v_1.7 stable cell line, HEK293 Nav1.7, RRID: CVCL_ZW92) (Hampl, Eberhardt, O'Reilly, & Lampert, 2016; Körner, Meents, Machtens, & Lampert, 2018). The mutants of hNa_v_1.7 were co‐transfected into this cell line, as was difopein and GFP as indicated in more detail below. Investigations of the currents of the Na_v_1.7 mutants alone were performed by transient overexpression of the vector in HEK293T cells.

#### 2.2.1 Experimental design

hNa_v_1.7/A1632E plasmids did not express as well as hNa_v_1.7/WT in HEK cells: When equal amount of cDNA was used, current density of hNav1.7/A1632E was significantly lower than of transiently transfected hNav1.7/WT (hNa_v_1.7/A1632E: 106.8 ± 89.3, *N* = 21; WT: 213.5 ± 143.1, *N* = 14. ^*^ *P* < 0.05). Transient double transfection (WT and A1632E) did not reveal enhancement of persistent current of the co‐transfected cells (WT: 1.8% ± 0.8%, *N* = 14; WT + hNa_v_1.7/A1632E: 3.4% ± 2.9%, *N* = 17; hNa_v_1.7/A1632E: 11.4% ± 10.2%, *N* = 21. ^*^ *P* < 0.05 for WT vs. A1632E and WT + A1632E vs. A1632E). This suggests that hNa_v_1.7/A1632E is not well expressed when transiently co‐transfected with WT Na_v_1.7. Thus, we chose to transfect the Na_v_1.7 stable cell line with the mutant, as a robust increase of persistent current in these conditions indicates co‐expression of WT and mutant channel.

Cells were grown in DMEM (Gibco‐Life Technologies, Carlsbad, CA, USA) containing 10% FBS (Gibco‐Life Technologies). For the Na_v_1.7 stable cell line, medium contained 1% Geneticin G418 (A&E Scientific, Marcq, Belgium) as a selection marker. Cells were incubated at 37°C in 5% CO_2_. Transfection was performed using Jet‐PEI reagent (POLYPLUS Transfection, Illkirch, France) and a total of 1.5 μg cDNA.

For single transfections of hNa_v_1.7 mutants, 1.25 μg plasmid was transfected into HEK293T cells, together with 0.25 μg of either pMax‐GFP or pIRES2‐EGFP‐difopein. Results from cells expressing hNa_v_1.7/R896Q and hNa_v_1.7/G375Afs were compared with those of the Na_v_1.7 stable cell line transfected with either 1.5 μg pMax‐GFP or pIRES2‐EGFP‐difopein as indicated. For co‐expression experiments, 1.25 μg hNa_v_1.7/A1632E, hNa_v_1.7/R896Q, or hNa_v_1.7/G375Afs was transfected into the Na_v_1.7 stable cell line, together with either 0.25 μg of pMax‐GFP or pIRES2‐EGFP‐difopein.

For experiments in which hNa_v_1.7/WT and hNa_v_1.7/A1632E were both transiently transfected into the HEK293T cell line, 0.625 μg of each plasmid (hNa_v_1.7 WT, hNa_v_1.7/A1632E) and 0.25 μg pMax‐GFP were used to obtain a total amount of 1.5 μg transfected cDNA. When hNa_v_1.7/WT or hNa_v_1.7/A1632E were transfected alone, 1.25 μg cDNA was combined with 0.25 μg pMax‐GFP.

### 2.3 Electrophysiology

Patch‐clamp and two‐electrode voltage‐clamp (TEVC) experiments were designed to compare persistent current and current density of channel constructs and their combination.

When transfection was performed, only HEK cells expressing GFP were used for whole‐cell patch‐clamp recordings at 27–60 h after transfection using an EPC 10 USB patch‐clamp amplifier (HEKA Elektronik, Lambrecht, Germany), with a sampling rate of 10 kHz. Patch pipettes made using a DMZ pipette puller (Zeitz‐Instrumente Vertrieb, Martinsried, Germany) and with a tip resistance in‐between 0.8 and 3.0 MΩ were used. All recordings were performed at room temperature (21 ± 2°C) with no correction for the liquid junction potential.

Patch‐clamp recordings used the following bath solution: 140 mM NaCl, 3 mM KCl, 1 mM MgCl_2_, 1 mM CaCl_2_, 10 mM HEPES, and 20 mM glucose, at pH 7.4 (adjusted using NaOH), and osmolarity of 310 ± 10 mOsm. The internal pipette solution contained 10 mM NaCl, 140 mM CsF, 1 mM EGTA, 10 mM HEPES, and 18 mM sucrose, at pH 7.33, and osmolarity of 310 ± 10 mOsm.

For HEK cell recordings, series resistance was ≤7 MΩ at all times for all cells and compensated to ≥65%. Leak current subtraction was performed online via a P/4 procedure. After reaching the whole‐cell configuration, cells were held at a holding potential of −120 mV for 3 min while they were pulsed with 0.1 Hz for stabilization of the inward current. Immediately afterwards, the current–voltage relationship was measured by stepwise 40 ms depolarizations from −90 mV to +40 mV in 10 mV increments.

For TEVC analysis of oocytes, hNa_v_1.7^His‐StrepIII^ and hNa_v_1.7/A1632E^His‐StrepII^ was expressed in *Xenopus laevis* oocytes. The oocytes were injected with capped and polyadenylated cRNA (in total 30–60 ng per oocyte) and maintained at 22°C in oocyte Ringer's solution (90 mM NaCl, 1 mM KCl, 1 mM CaCl_2_, 1 mM MgCl_2_, and 10 mM HEPES/NaOH pH 7.4) containing 50 μg·ml^−1^ gentamycin. For co‐expression of hNa_v_1.7/WT and hNa_v_1.7/A1632E, cRNAs encoding the hNa_v_1.7/WT and hNa_v_1.7/A1632E mutant were injected in a 1:1 ratio (w/w). Currents were recorded at room temperature (21–23°C) in oocyte Ringer's solution 1–3 days following cRNA injection by conventional TEVC electrophysiology using a Turbo TEC‐05 amplifier (NPI Electronic GmbH, Germany) controlled by the PatchMaster software (HEKA Elektronik GmbH, Germany Patchmaster, RRID:SCR_000034) as detailed previously (Hausmann et al., 2014; Kaluza et al., 2018). Intracellular microelectrodes were pulled with a PC‐100 pipette puller (Narishige, Japan) from borosilicate glass capillaries and filled with 3 M KCl, yielding tip resistances of 0.3–0.5 MΩ. Whole‐cell Na^+^ currents were low‐pass filtered at 2 kHz and sampled at 100 kHz. Each oocyte was clamped at a holding potential of −100 mV for at least 3–5 min to ensure recovery from slow inactivation before current recordings, and pulse protocols were started. Transients and leak currents were subtracted online by using the P/N (P/8) procedure implemented in the PatchMaster software. The current–voltage relationship was determined from a holding potential of −100 mV by stepwise 50 ms depolarizations from −90 mV to +40 mV in 10 mV increments with an interval of 5 s.

In patch‐clamp and TEVC experiments, the persistent current was determined between 34 and 39.6 ms of each test‐pulse and normalized to the transient peak inward current of the same cell. To exclude effects of varying cell size and current density (Figure S5 Supplementary Information) on persistent current, we analysed persistent current as a percentage of the maximum peak current for each cell. The maximum persistent current for each cell was then used for comparison. In order to compare the peak inward current from cells of different sizes, we normalized the peak current of each cell to the cell capacitance and calculated current density as pA/pF in HEK cells.

### 2.4 PCR

PCR was performed with RNA extracted from untransfected HEK293T cells, the Na_v_1.7 stable cell line and *X.* *laevis* oocytes. RNA was extracted using a Nucleospin RNA kit (Macherey‐Nagel, Düren, Germany), according to the manufacturer's instructions and cDNA was synthesized with a SensiFAST cDNA synthesis kit (Bioline, London, UK). Testing for the different 14‐3‐3 isoforms was done using a set of seven different human primers (produced by Eurofins Genomics, Ebersberg, Germany; Tables S1 and S2), along with Taq DNA polymerase, Thermopol Buffer, and dNTPs (New England BioLabs, Frankfurt am Main, Germany). The PCR protocol included 35 cycles and an annealing temperature of 52°C.

### 2.5 Biochemical analysis of hNa_v_1.5^GFP^ and hNa_v_1.7^GFP^ expressed in *X. laevis* oocytes

The procedures for housing, anaesthesia, and surgical ovarectomy of *X. laevis* frogs were approved by the relevant Animal Welfare Authority (LANUV, Recklinghausen, Germany; reference nos. 84‐02.04.2014.A366 and 81‐02.04.2019.A355), in compliance with Directive 2010/63/EU of the European Parliament. Animal studies are reported in compliance with the ARRIVE guidelines (Kilkenny et al., 2010) and the editorial on reporting animal studies (McGrath & Lilley, 2015) as well as with the recommendations made by the *British Journal of Pharmacology.*

For biochemical characterization, hNa_v_1.7 was expressed in collagenase‐defolliculated *X. laevis* oocytes (Dumont stages V and VI) as previously described (Stolz et al., 2015). The oocytes were injected with capped and polyadenylated cRNA (30–60 ng per oocyte) and maintained at 19°C in oocyte Ringer's solution containing 50 μg·ml^−1^ gentamycin. At day 2 post‐cRNA injection, the oocytes were homogenized in ice‐cold 0.1 M sodium phosphate, pH 8.0 (10 μl per oocyte) containing 1 mM tris(2‐carboxyethyl)phosphine HCl and protease inhibitors (pepstatin, leupeptin, antipain, and Pefabloc SC) plus one of the following non‐ionic detergents: digitonin (water‐soluble quality; Serva, Heidelberg, Germany), DDM (AppliChem, Darmstadt, Germany), glyco‐diosgenin, or NG310 (Anatrace, Maumee, OH, USA). Detergent concentrations are indicated in the figures. After two clearing spins at 10,000× *g* for 15 min each, aliquots of the supernatant were resolved by hrCN‐PAGE (Wittig, Braun, & Schagger, 2006) and clear SDS‐urea‐PAGE (Fallah et al., 2011; Stolz et al., 2015). Gels were destained by overnight incubation in acetonitrile/ammonium carbonate and washed twice in 0.1 M sodium phosphate pH 8.0 (Fallah et al., 2011; Stolz et al., 2015); wet gels were then scanned on a Typhoon fluorescence scanner (GE Healthcare). Figures were prepared with halftone images using ImageQuant TL 8.2 software (GE Healthcare; Image Quant TL, RRID:SCR_018374) for contrast adjustments, Adobe Photoshop CS 8.0 (Adobe Photoshop, RRID:SCR_014199) for level adjustment and cropping, and Microsoft PowerPoint 2000 for labelling.

### 2.6 Molecular dynamics simulations

All‐atom molecular dynamics (MD) simulations of the hNa_v_1.7 α/β1 subunit complex (Protein Data Bank [PDB] ID: 6J8I) (Shen et al., 2019) were carried out using the CHARMM36m force field with the TIP3P water model (Huang et al., 2017). The simulation box contained an equilibrated 1‐palmitoyl‐2‐oleoyl‐sn‐glycero‐3‐phosphocholine lipid bilayer, surrounded by 250 mM NaCl aqueous solution. The DIII–DIV linker containing the IFM motif was removed from the channel structure, and Na_v_1.7 was modelled using the residue ranges 114–417, 715–961, and 1,164–1,757 for the α subunit and 20–192 for the β1 subunit (isoform 3 residue numbering). The complex was embedded into the bilayer using *g_membed* (Wolf, Hoefling, Aponte‐Santamaria, Grubmuller, & Groenhof, 2010). Glu180 and Asp1256 were assigned a protonated state, and all other amino acids were modelled in their default ionization state to reflect the most probable state at neutral pH based on pKa calculations using PROPKA 3.1 (Olsson, Sondergaard, Rostkowski, & Jensen, 2011; Sondergaard, Olsson, Rostkowski, & Jensen, 2011).

We performed MD simulations using GROMACS 2018 (Abraham et al., 2015; GROMACS, RRID:SCR_014565) in the NPT ensemble with periodic boundary conditions and an integration time step of 2 fs. Temperature was maintained at 310 K using the velocity‐rescaling thermostat; pressure was maintained at 1 bar using the semi‐isotropic Parrinello–Rahman barostat as described (Machtens et al., 2015). Lennard–Jones interactions were truncated at 12 Å with a force switch smoothing function from 10 to 12 Å. Electrostatic interactions were calculated using the particle mesh Ewald method and a real space cut‐off of 12 Å. The simulation systems of WT and A1632E Na_v_1.7 were equilibrated with position restraints on the protein heavy atoms for 500 ns, followed by ~20 ns with backbone‐only position restraints, and 200‐ns production runs without any restraints. We used MODELLER (MODELLER, RRID:SCR_008395) to insert the A1632E side chain substitution (Webb & Sali, 2016).

Water densities were calculated from the production runs in the time window of 10–200 ns using GROmaps (Briones, Blau, Kutzner, de Groot, & Aponte‐Santamaria, 2019) and visualized by mapping water densities onto the water‐accessible protein surface of the IFM‐binding pocket. PyMOL (PyMOL, RRID:SCR_000305) was used to render all molecular images.

### 2.7 Sequence alignment

The putative dimerization sites of hNa_v_1.5 channels (Clatot et al., 2017) were aligned with different human VGSC subtypes using UniProt (Universal Protein Resource, RRID:SCR_002380) and BLAST (BLASTX, RRID:SCR_001653) (Altschul, Gish, Miller, Myers, & Lipman, 1990; The UniProt Consortium, 2019).

### 2.8 Data and statistical analysis

Fitmaster software (HEKA Elektronik, Lambrecht, Germany; FITMASTER, RRID:SCR_016233) and Igor Pro software (WaveMetrics, Portland OR, USA; IGOR Pro, RRID:SCR_000325) were used for data analysis. Statistical analysis was performed with Prism 7 (GraphPad, San Diego CA, USA; GraphPad Prism, RRID:SCR_002798). Experimental units used in the statistics were the numbers of single cells which provided only one set of recordings.

The peak current density or the maximum persistent current of each cell in the different groups were tested for Gaussian distribution using a D'Agostino Pearson test. Mann–Whitney tests were applied for non‐parametric testing and unpaired *t*‐tests for parametric testing. One‐way ANOVA was used for comparing more than two groups. If *F* was statistically significant (*P* < 0.05), a Bonferroni post hoc test was applied. As only the maximum current density/persistent current of each cell was compared, we chose this form of testing as appropriate, as we used parameters that were not matched. In figures where the I/V protocols are displayed as persistent current per voltage step, these data were not statistically analysed but are shown displayed for graphical illustration. Standard deviation (SD) was used to display data. The *N*'s for all data sets are indicated in the figure legends. The results and the exact *N* for each data point of the I/V protocols can be found in the Tables S3–S14, Supplementary Information.

For estimation of the number of cells needed per experiment, we performed an a priori analysis for experiments where two groups—assuming normal distribution—were compared (*d* = 0.8, *α* = 0.05, power = 0.7, *N* = 21 per group), for experiments with three groups (*f* = 0.4, *α* = 0.05, power = 0.7, *N* = 18 per group), and for experiments with six groups (*f* = 0.4, *α* = 0.05, power = 0.7, *N* = 12 per group). We referred to the calculated results from this analysis and other studies with similar investigations (Cox et al., 2006; Estacion et al., 2008; Meents et al., 2018). Studies were designed to generate groups of equal size. Inequality in group size may occur due to exclusion of individual cells during data analysis (e.g., high series resistance).

Declared group size is the number of independent values, and statistical analysis was done using these independent values. Statistical analysis was only undertaken for groups with *n* > 5. As the difference in the cells regarding the occurrence of persistent current was very obvious at first sight, blinding was hard to achieve, and thus, observer blinding was not performed.

Sample randomization was ensured by measuring cells from more than one dish per group of several transfections from varying passages and by recording cells from different groups (e.g., WT and mutant) on each experimental day.

Outliers were not excluded from analysis except in Figure 5d. One cell of the co‐transfection of hNa_v_1.7/WT and hNa_v_1.7/R896Q presented a current density of 882.76 pA/pF which is far above the current density of the other cells and was checked for significance using Grubb's test (GraphPad QuickCalcs, San Diego CA, USA) and excluded from analysis since the difference to the other values was so large. As a consequence, all groups in Figure 5d were checked for significant outliers using Grubb's test, leading to the exclusion of one outlier in the group hNa_v_1.7/WT + hNa_v_1.7/R896Q + difopein and one outlier in the group hNa_v_1.7/WT + difopein.

The threshold for statistical significance was set to *P* < 0.05. The data and statistical analysis comply with the recommendations of the *British Journal of Pharmacology* on experimental design and analysis in pharmacology (Curtis et al., 2018).

### 2.9 Materials

The materials used in these experiments were supplied as follows:DDM from AppliChem (Darmstadt, Germany); digitonin from Serva (Heidelberg, Germany); Dpn I restriction endonuclease and Phusion high‐fidelity DNA polymerase from New England Biolabs (Frankfurt, Germany); Dulbecco's modified Eagle's medium and FBS from Gibco‐Life Technologies (Carlsbad, CA, USA); *Escherichia coli* cells, Taq DNA polymerase, Thermpol Buffer and dNTPs from New England Biolabs (Frankfurt, Germany); Gateway PCR Cloning System and In‐Fusion HD Cloning from Takara Bio Europe (Göteborg, Sweden); geneticin and G418 from A&E Scientific (Marcq, Belgium); Glycol‐diosgenin and NG310 from Anatrace (Maumee, OH, USA); Nucleospin RNA kit from Macherey‐Nagel, Düren, Germany); pIRES2‐EGFP‐difopein from BioCat (Heidelberg, Germany); pMax‐GFP from Lonza (Basel, Switzerland); QuikChange primers, megaprimer‐PCR primers, 14‐3‐3 primers from Eurofins Genomics (Ebersberg, Germany); salts (NaCl, KCl, CaCl_2_, MgCl_2_), HEPES, NaOH for oocyte Ringer solution from Sigma‐Aldrich/Merck (Darmstadt Germany); SensiFast cDNA synthesis kit from Bioline (London, UK).

#### 2.10 Nomenclature of targets and ligands

Key protein targets and ligands in this article are hyperlinked to corresponding entries in the IUPHAR/BPS Guide to PHARMACOLOGY (http://www.guidetopharmacology.org) and are permanently archived in the Concise Guide to PHARMACOLOGY 2019/20 (Alexander et al., 2019).

### 3 Results

#### 3.1 hNa_v_1.7/A1632E exhibits impairment of fast inactivation—Evidence for an allosteric mechanism

**FIGURE 1:**
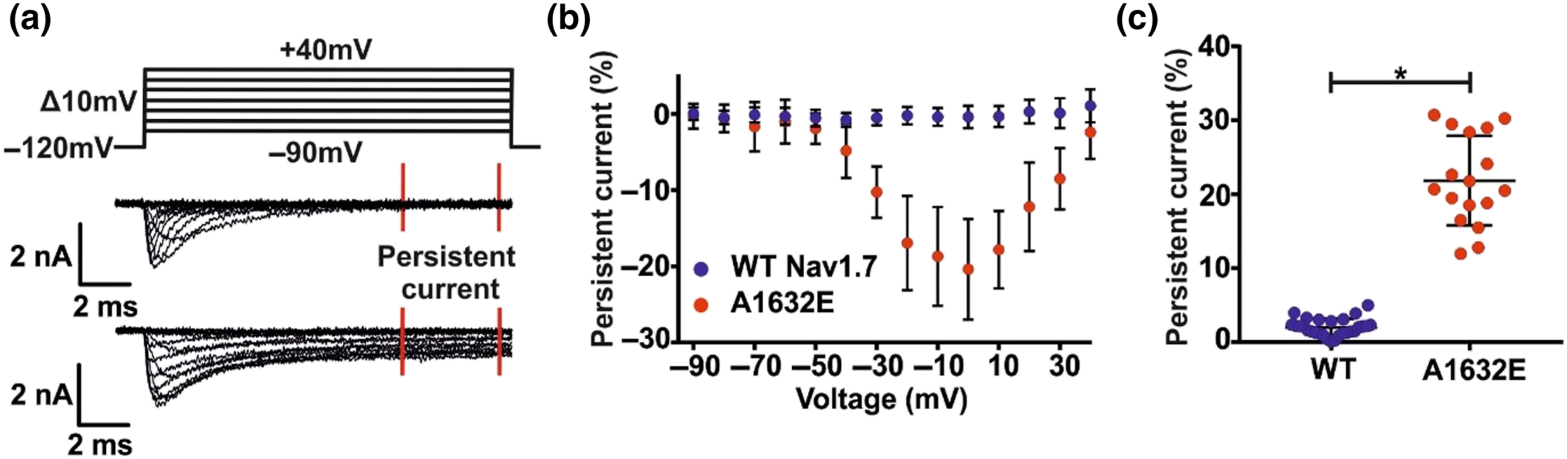
Expression of hNa_v_1.7/A1632E channels causes a prominent persistent current compared to that in a Na_v_1.7 channel stable cell line. (a) Representative traces of hNa_v_1.7/WT (middle panel) and hNa_v_1.7/A1632E (bottom panel) sodium currents. The persistent current was measured in the indicated range (between the red bars). The voltage protocol is shown in the top panel. Concentration of transfected cDNA: WT: Na_v_1.7 stable cell line; A1632E: 1.25 μg hNa_v_1.7/A1632E + 0.25 μg GFP (HEK293T cells). (b) Relative persistent current of HEK cells expressing the indicated hNa_v_1.7 channels. The measured persistent current at each voltage step was normalized to the peak inward current of the cell. Data were not statistically analysed. For means, SD, and *N* for each data point, please refer to Tables S3‐S4. (c) Maximal relative persistent current of each cell as used for statistical comparison. Data shown are individual values with means ± *SD*; hNa_v_1.7/WT, *N* = 28; hNa_v_1.7/A1632E, *N* = 17. Difference of means: 19.8%, 95% CI of difference of means: {17.5; 22.2}. ^*^ *P* < 0.05, significantly different as indicated

Using patch‐clamp of HEK cells overexpressing the pain‐linked mutation hNa_v_1.7/A1632E, we confirmed its reported prominent persistent current (Figure 1), suggesting an impaired fast inactivation as a major disease‐linked gating change (Dib‐Hajj, Yang, Black, & Waxman, 2013).

**FIGURE 2:**
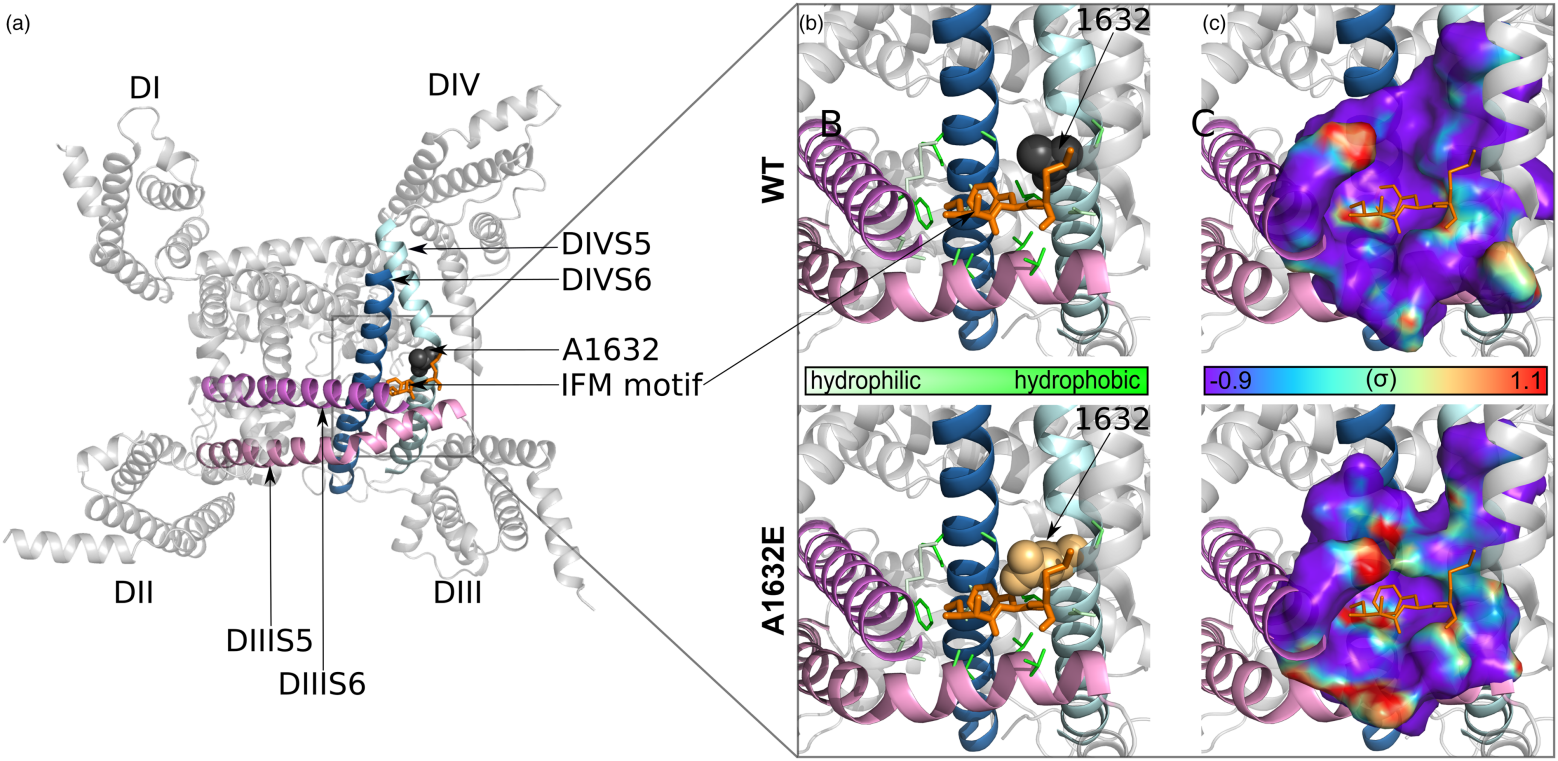
The A1632E mutation impedes binding of the IFM motif via steric repulsion and hydrophobic mismatch. (a) Overview of the cryo‐EM structure of the hNa_v_1.7 α‐subunit from the intracellular side (PDB ID: 6J8I). Spheres, A1632 of DIV S5; orange sticks, the IFM motif. (b) Close‐up view of the IFM‐binding pocket, a hydrophobic cavity formed by residues from the DIII S4–S5 linker, DIII S5, DIII S6, DIV S5, and D‐IV S6 in both WT (upper) and A1632E Na_v_1.7 (lower). Thin sticks, pocket‐forming residues; thick sticks, IFM‐motif residues; spheres, residue 1632. Intensity of green colour indicates the hydrophilicity/hydrophobicity. (c) Solvent‐accessible surface of the IFM‐binding pocket from the same perspective as shown in (b). Water densities from unguided 200‐ns all‐atom MD simulations of WT and A1632E Na_v_1.7 (with the DIII ‐ DIV linker removed) were mapped onto the protein surface, with mapping strength indicated by the colour‐scale bar. The IFM motif was not present in these simulations, but its position in the IFM‐bound structure is indicated by orange lines

The recently published cryo‐EM structures of hNa_v_1.4 and hNa_v_1.7 channels revealed that the IFM motif needed for fast inactivation is tightly bound to a hydrophobic cavity formed by the S5 and S6 of domains III and IV and the DIII S4–S5 linker at the side of the pore (Pan et al., 2018; Shen et al., 2019). As A1632, located on DIV S5, is in proximity of this cavity (Figure 2a), substitution of A1632 by glutamate might affect binding of the IFM motif. To test this hypothesis, we modelled the A1632E substitution in the hNa_v_1.7 structure. The large glutamate side chain was shown to protrude into the binding pocket and could therefore cause steric repulsion of the IFM motif (Figure 2b). However, as substitution of A1632 with aspartate (which is smaller than glutamate) has been shown to induce even larger persistent currents that that with A1632E (Eberhardt et al., 2014), steric repulsion of the IFM motif is unlikely to be the only explanation for these effects in the mutant channels.

Tight binding of the IFM motif requires a strongly hydrophobic binding pocket, which could be disrupted by the increased attraction of water molecules into the pocket caused by the hydrophilic A1632E substitution. We therefore performed unguided all‐atom molecular dynamics simulations to measure water accessibility of the empty IFM‐binding pocket. Whereas the hydrophobic nature of most amino acid side chains lining the wild type (WT) binding pocket results in rather low water accessibility, the A1632E mutation increased hydration at the IFM‐binding position (Figure 2c). Thus, inhibition of IFM motif binding via both steric repulsion and hydrophobic mismatch with the binding pocket contribute to the mutant phenotype of A1632E. Taking into account the recently proposed allosteric inactivation mechanism (Yan et al., 2017), the larger glutamic acid and increased hydration of the binding pocket in the hNa_v_1.7/A1632E mutant will impair binding of the IFM motif, inhibit fast inactivation, and result in a persistent current. Thus, in the mutant channel, DIV S6 may be less prone to moving into the ion permeation pathway, and the pore may therefore not inactivate properly.

#### 3.2 Voltage‐gated sodium channel Na_v_1.7 dimerizes

**FIGURE 3:**
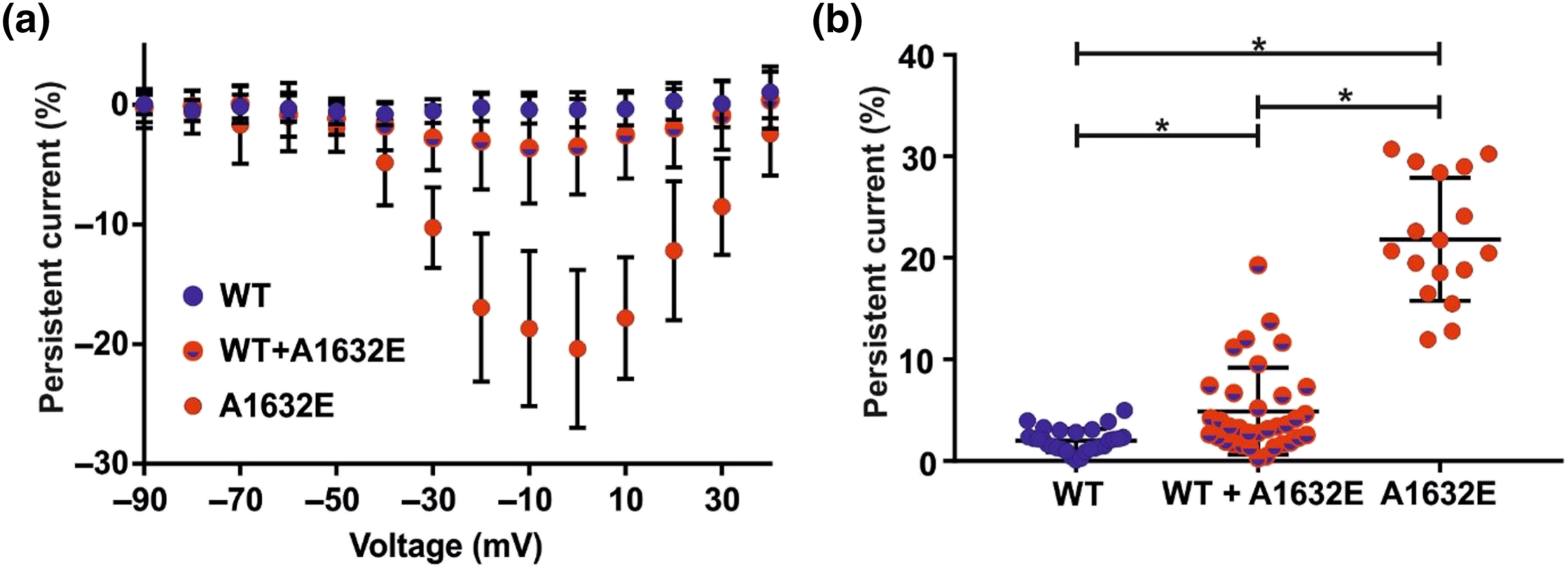
Co‐expression of hNa_v_1.7/WT and hNa_v_1.7/A1632E in a Na_v_1.7 stable cell line disproportionally affects persistent current. (a) Relative persistent current of HEK cells expressing the indicated hNa_v_1.7 channels. The measured persistent current at each voltage step was normalized to the peak inward current of the cell. Concentration of transfected cDNA: WT: Na_v_1.7 stable cell line; WT + A1632E: 1.25 μg hNa_v_1.7/A1632E + 0.25 μg GFP (Na_v_1.7 stable cell line); A1632E: 1.25 μg hNa_v_1.7/A1632E + 0.25 μg GFP (HEK293T cells). Data were not statistically analysed. For mean, *SD*, and *N* of each data point, please refer to Tables S5‐ S7. (b) Maximal relative persistent of each cell as used for statistical analysis. Data shown are individual values with means ± *SD;* hNa_v_1.7/WT, *N* = 28; hNa_v_1.7/WT + hNa_v_1.7/A1632E, *N* = 35; hNa_v_1.7/A1632E, *N* = 17. Difference of means: hNa_v_1.7/WT − hNa_v_1.7/WT + hNa_v_1.7/A1632E: −2.9%, 95% CI of difference of means: {−5.4; −0.4}; hNa_v_1.7/WT − hNa_v_1.7/A1632E: −19.8% {−22.9; −16.8}; hNa_v_1.7/WT + hNa_v_1.7/A1632E − hNa_v_1.7/A1632E: −16.9% {−19.8; −14.0}. ^*^ *P* < 0.05, significantly different as indicated

**FIGURE 4:**
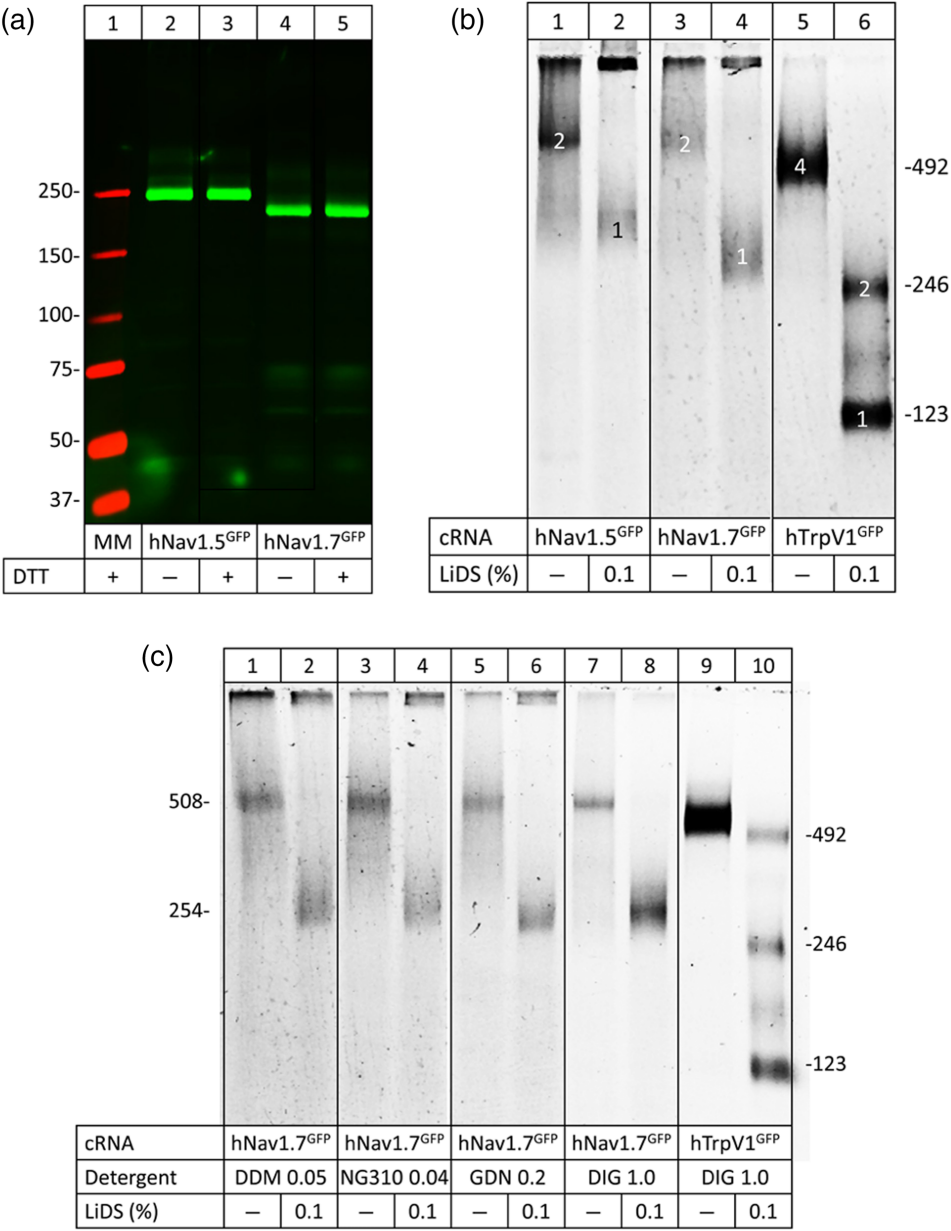
Na_v_1.5 and Na_v_1.7 migrate as non‐covalent homodimers under native PAGE. (a) SDS‐urea‐PAGE of hNa_v_1.5 and hNa_v_1.7 in the presence or absence of the reducing agent DTT. (b) and (c) hrCN‐PAGE of hNa_v_1.5and hNa_v_1.7 with various detergents used to turn dimers into monomers. The indicated channel proteins were extracted from *Xenopus laevis* oocytes with digitonin (a, b) or one of the indicated detergents (c), resolved by hrCN‐PAGE, and the GFP tags were visualized by Typhoon fluorescence scanning. Protein migration is shown under native conditions and after partial denaturation (1‐h incubation with 0.1% LiDS at 37°C). Numbers in the right margins in (b) and (c) indicate the sequence‐calculated masses (protomers to tetramers) of the partially denatured hTrpV1‐GFP channel. Numbers in the left margin in (c) correspond to the sequence‐calculated masses of the hNa_v_1.7‐GFP protomer and homodimer. NG310, lauryl maltose neopentyl glycol; GDN, glyco‐diosgenin; DIG, digitonin

Recent studies on channel dimerization in hNa_v_1.5 channels suggested that mutants can have an effect on the WT channel when co‐transfected (Clatot et al., 2017, 2018). When A1632E was co‐expressed with WT channels, its persistent current was disproportionally reduced (Figure 3a,b). To test if hNa_v_1.7 channels also form functionally coupled dimers, we solubilized hNa_v_1.7^GFP^‐expressing *X. laevis* oocytes in the non‐ionic detergent digitonin and resolved the proteins by sodium dodecyl sulfate (SDS)‐urea‐PAGE and high resolution clear native (hrCN)‐PAGE. hNa_v_1.5^GFP^ was used as the positive control. In the SDS‐PAGE gel, hNa_v_1.5^GFP^ and hNa_v_1.7^GFP^ migrated with apparent molecular masses of 250 and 220 kDa, respectively, in both, the non‐reduced and reduced states. Reasonable agreement with the sequence‐calculated masses of 256 and 254 kDa, respectively (Figure 4a), argues against the existence of disulfide‐bonded VGSC dimers but not against non‐covalently bound dimers.

When the same samples were resolved by hrCN‐PAGE, hNa_v_1.5^GFP^ and hNa_v_1.7^GFP^ migrated under non‐denaturing conditions with apparent molecular masses of about 560 kDa (Figure 4b, lanes 1 and 3, labelled with “2”). We compared the migration under denaturing conditions of these VGSC proteins with hTrpV1^GFP^, a 492 kDa homotetramer (Julius, 2013) consisting of four protomers with a calculated mass of 123 kDa each (Figure 4b, lane 5). Treatment with a low concentration (0.1%) of the denaturant lithium dodecyl sulfate (LiDS) resulted in dissociation of hTrpV1^GFP^ homotetramers into homodimers and protomers with calculated masses of 246 and 123 kDa, respectively (Figure 4b, lane 6). The VGSC proteins also dissociated into faster migrating species. Comparison with hTrpV1^GFP^ showed that hNa_v_1.5^GFP^ and hNa_v_1.7^GFP^ protein migrated at ~290 kDa in the presence of LiDS (Figure 4b, lanes 2 and 4, indicated by “1”). These results suggest that both hNa_v_1.5 and hNa_v_1.7 channels have a strong tendency to assemble into homodimers in *X. laevis* oocytes.

To investigate the effect of different solubilization detergents on oligomerization, we solubilized hNa_v_1.7^GFP^‐expressing oocytes in four non‐ionic detergents: lauryl maltose neopentyl glycol (NG310), glyco‐diosgenin, digitonin, and *n*‐dodecyl β‐d‐maltoside (DDM). As with LiDS treatment, hrCN‐PAGE resulted in monomers and homodimers in the presence or absence of detergent, respectively (Figure 4c). Thus, our data clearly indicate that similar to hNa_v_1.5, hNa_v_1.7 channels are able to form homodimers.

**FIGURE 5:**
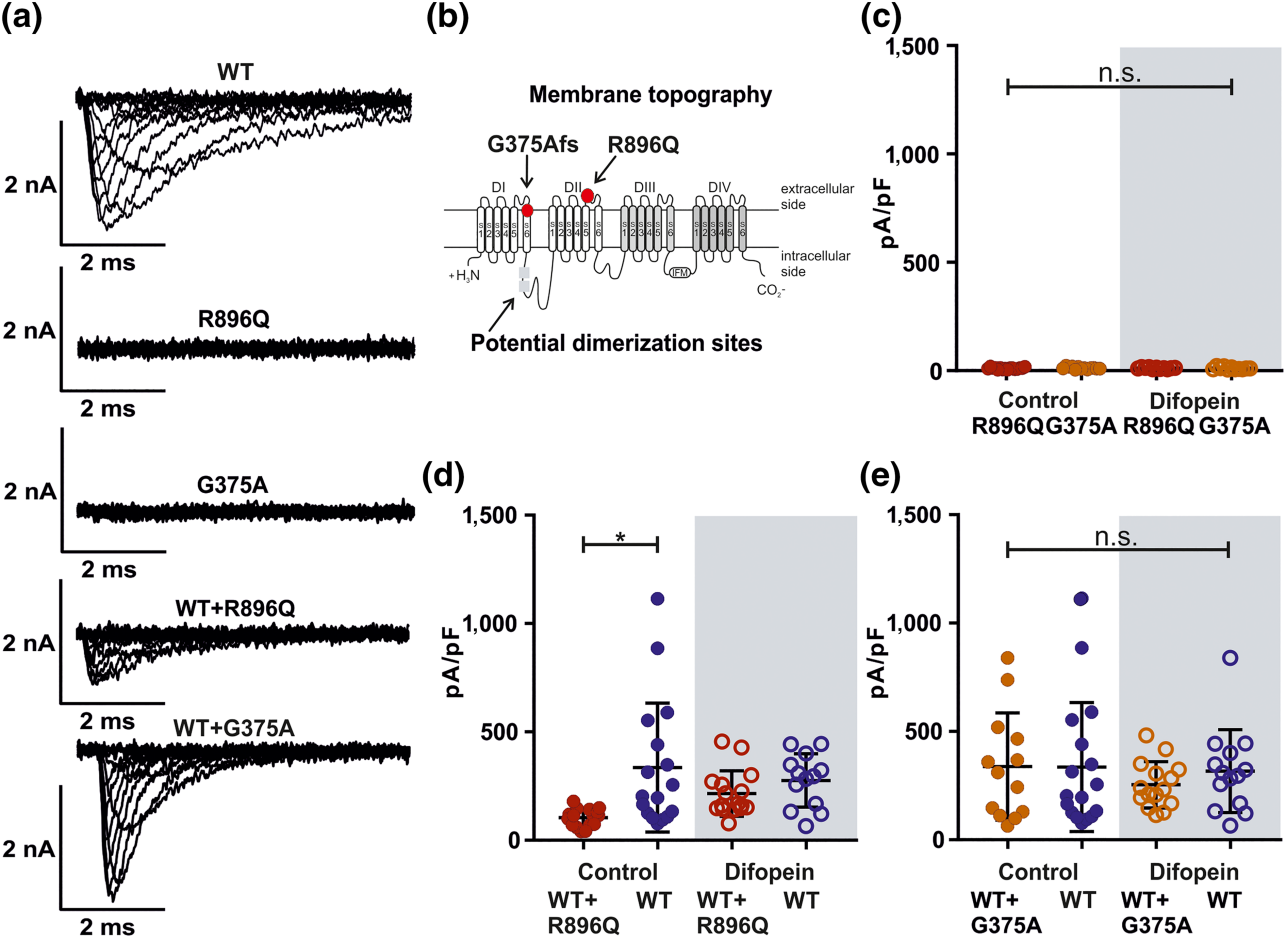
Loss‐of‐function mutations support dimerization of hNa_v_1.7 channels in a Na_v_1.7stable cell line. (a) Representative traces of (top to bottom): hNa_v_1.7/WT, hNa_v_1.7/R896Q, hNa_v_1.7/G375Afs, hNa_v_1.7/WT + hNa_v_1.7/R896Q, and hNa_v_1.7/WT + hNa_v_1.7/G375Afs. Concentration of transfected cDNA: WT: Na_v_1.7 stable cell line + 1.5 μg GFP or difopein; WT + R896Q ± difopein: 1.25 μg hNa_v_1.7/R896Q + 0.25 μg GFP or difopein (Nav1.7 stable cell line); WT + G37Afs ± difopein: 1.25 μg hNav1.7/G375Afs + 0.25 μg GFP or difopein (Nav1.7 stable cell line); R896Q ± difopein: 1.25 μg hNa_v_1.7/R896Q + 0.25 μg GFP or difopein (HEK293T cells); G375Afs ± difopein: 1.25 μg hNa_v_1.7/G375Afs + 0.25 μg GFP or difopein (HEK293T cells). (b) Schematic representation of hNa_v_1.7 showing the sites mutated in hNa_v_1.7/G375Afs, hNa_v_1.7/R896Q (red dots) and the possible dimerization site in the linker region between DI and DII (grey rectangle). (c) Peak current density in pA/pF was used for statistical analysis. Data shown are individual values with means ± *SD;* hNa_v_1.7/R896Q: 10.8 ± 4.3, *N* = 16; hNa_v_1.7/G375Afs: 12.4 ± 5.8, *N* = 13; hNa_v_1.7/R896Q + difopein: 11.9 ± 3.4, *N* = 11; hNa_v_1.7/G375Afs + difopein: 11.3 ± 5.6, *N* = 11.n.s. = not significant as indicated. (d) Peak current density in pA/pF was used for statistical analysis. Data shown are individual values with means ± *SD;* hNa_v_1.7/WT + hNa_v_1.7/R896Q, *N* = 16; hNa_v_1.7/WT, *N* = 17; hNa_v_1.7/WT + hNa_v_1.7/R896Q + difopein, *N* = 16; hNa_v_1.7/WT + difopein, *N* = 13. ^*^ *P* < 0.05. Difference of means: hNa_v_1.7/WT − hNa_v_1.7/WT + hNa_v_1.7/R896Q: −231.4, 95% CI of difference of means: {−398.7; −64.1}; hNa_v_1.7/WT + difopein − hNa_v_1.7/WT + hNa_v_1.7/R896Q + difopein: −60.1 {−239.4; 119.3}. ^*^ *P* < 0.05, significantly different, n.s. = not significant, as indicated. (e) Peak current density in pA/pF was used for statistical analysis: Data shown are individual values with means ± *SD.* hNa_v_1.7/WT + hNa_v_1.7/G375Afs, *N* = 13; hNa_v_1.7/WT, *N* = 17; hNa_v_1.7/WT + hNa_v_1.7/G375Afs + difopein, *N* = 15; hNa_v_1.7/WT + difopein, *N* = 14. Difference of means: hNa_v_1.7/WT − hNa_v_1.7/WT + hNa_v_1.7/G375Afs: 2.4, 95% CI of difference of means: {−224.8; 229.6}; hNa_v_1.7/WT + difopein − hNa_v_1.7/WT + G375Afs + difopein: −62.6 {−291.7;166.6}. n.s. = not significant as indicated

To confirm the effects of channel dimerization on a functional level in hNa_v_1.7 channels, we compared two loss‐of‐function mutants reported to occur in CIP patients: the single point mutation hNa_v_1.7/R896Q and truncation mutation hNa_v_1.7/G375Afs (Cox et al., 2010; Shorer, Wajsbrot, Liran, Levy, & Parvari, 2014). When expressed in HEK cells, no current was detected for either protein (Figure 5a,c). hNa_v_1.7/G375Afs is truncated at a position before the two proposed dimerization sites (Figure 5b) (Clatot et al., 2017), suggesting that the mutant channel would not be able to interact with hNa_v_1.7/WT. In contrast, hNa_v_1.7/R896Q is thought to form complete channels and should thus form dimers with hNa_v_1.7/WT (see Figure S1, Supplementary Information, for an alignment of the putative dimerization sites).

**FIGURE 6:**
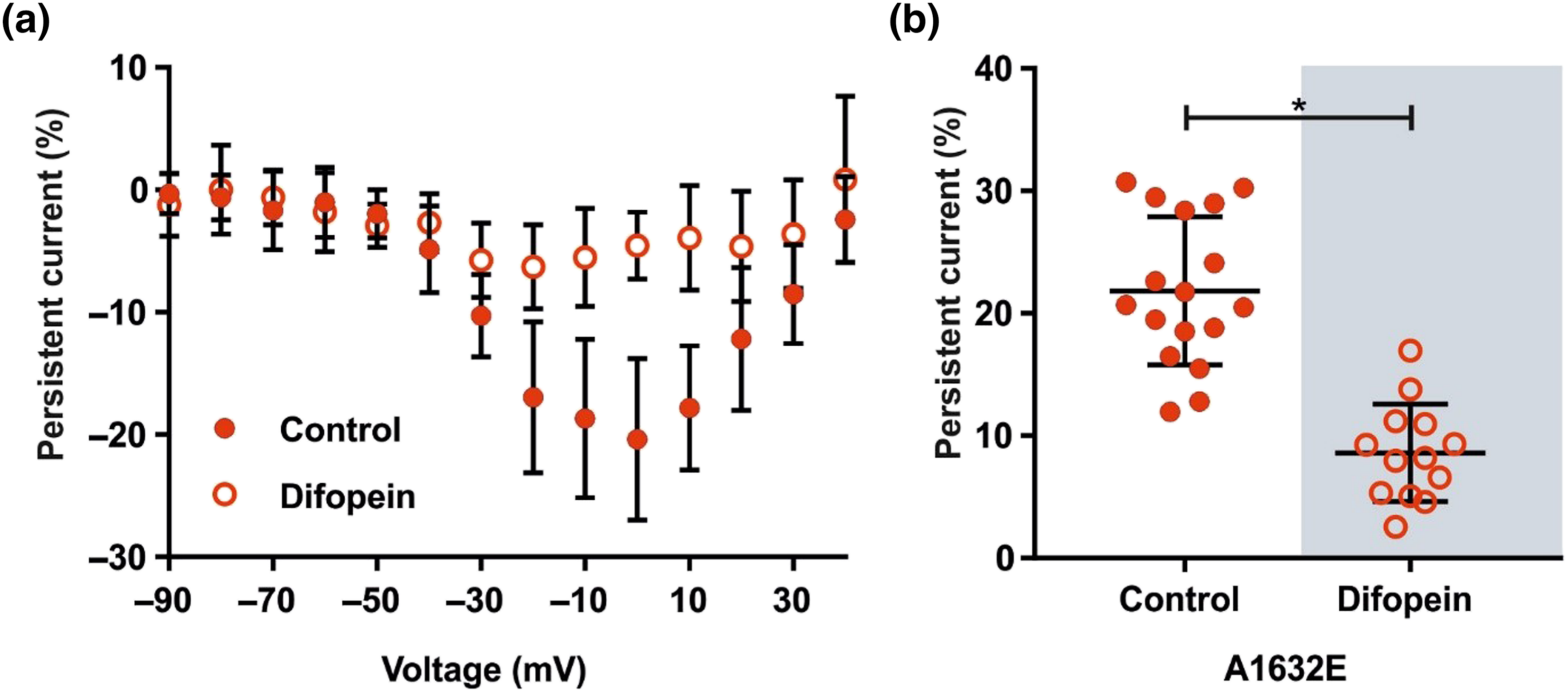
Difopein reduces the size of the persistent current of hNa_v_1.7/A1632E channels in HEK293T cells. (a) Relative persistent current of HEK cells expressing the hNa_v_1.7/A1632E channel in absence or presence of difopein. The measured persistent current at each voltage step was normalized to the peak inward current of the cell. Concentration of transfected cDNA: A1632E: 1.25 μg hNa_v_1.7/A1632E + 0.25 μg GFP or difopein (HEK293T cells). Data were not statistically analysed. For mean, *SD* and *N* of each data point, please refer to Tables S8‐S9. (b) Maximal relative persistent current of each cell is shown, as it was used for statistical comparison. Data shown are individual values with means ± *SD*; hNa_v_1.7/A1632E, *N* = 17; hNa_v_1.7/A1632E + difopein, *N* = 13. Difference of means: 13.2%, 95% CI of difference of means: {−17.2; −9.3}. ^*^ *P* < 0.05, significantly different as indicated

Co‐expression of hNa_v_1.7/R896Q in the hNa_v_1.7 stable cell line led to a significantly reduced current density compared with stably transfected hNav1.7/WT alone (Figure 5d), suggesting an interference between those two channels. Functional coupling of dimers is suggested to be mediated by binding of 14‐3‐3, which can be inhibited using difopein (Clatot et al., 2017) (see Figure S2, Supplementary Information for expression of 14‐3‐3 in HEK cells). Co‐expression of difopein abolishes the previously significant difference in current density between hNa_v_1.7/WT alone and hNa_v_1.7/WT co‐expressed with hNa_v_1.7/R896Q (Figure 5d). Difopein did not alter the current density of hNa_v_1.7/WT alone (Figure 5d).

In contrast, co‐expression of the WT with the truncation mutant hNa_v_1.7/G375Afs did not alter the current density of hNa_v_1.7/WT, with or without difopein (Figure 5e). This result suggests that the truncated hNa_v_1.7/G375Afs is unlikely to dimerize with full‐length hNa_v_1.7/WT channels (although lack of expression cannot be excluded, see Section 4). Thus, the potential dimerization sites are likely to reside between the positions of the two mutations, possibly in the DI–DII linker, similar to the proposed dimerization site of hNa_v_1.5 (Clatot et al., 2017).

As our data suggest functional dimerization of hNa_v_1.7, we set out to investigate its effect on the allosteric inactivation mechanism: We co‐expressed the difopein peptide with hNa_v_1.7/A1632E in order to suppress functional dimerization. The presence of difopein reduced the persistent current of hNa_v_1.7/A1632E by more than half (Figure 6).

To provide further support for our findings, we performed TEVC experiments using *X. laevis* oocytes expressing either the hNa_v_1.7/WT or hNa_v_1.7/A1632E or both. *X. laevis* oocytes offer the unique advantage that the delivery of two distinct cRNAs at an exactly defined molar ratio into every cell can be controlled by microinjection (Kowalski et al., 2015; Terhag, Cavara, & Hollmann, 2010).

**FIGURE 7:**
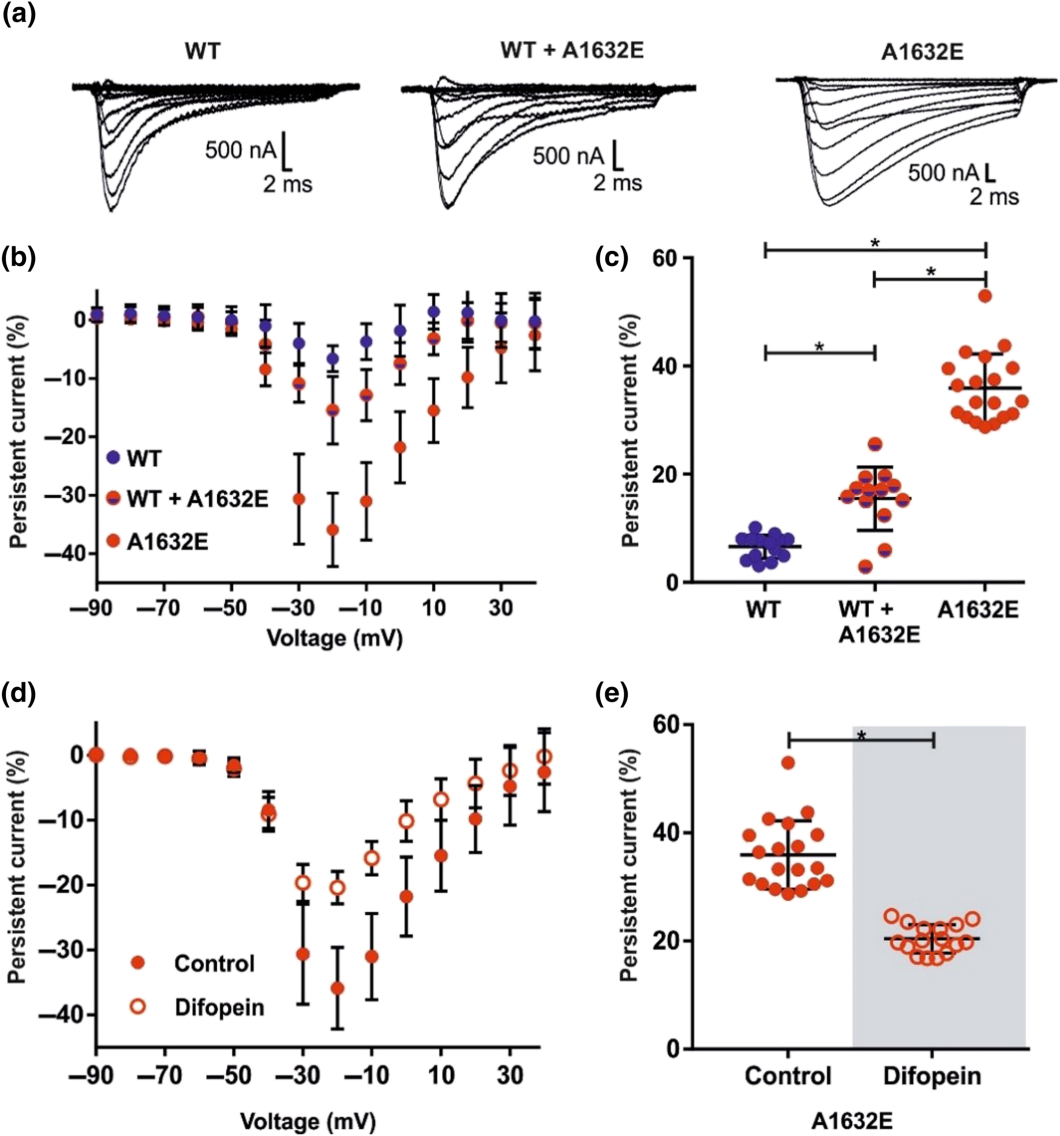
Expression of hNa_v_1.7/WT and hNa_v_1.7/A1632E channels in *Xenopus laevis* oocytes. (a) Representative current traces of *X. laevis* oocytes expressing hNa_v_1.7/WT (left panel), hNa_v_1.7/A1632E (right panel) or the combination of WT and A1632E (middle panel) elicited by stepwise depolarizations from −90 mV to +40 mV in 10 mV increments (from a holding potential of −100 mV). (b) Relative persistent current of oocytes expressing the indicated hNav1.7 channels. The measured persistent current at each voltage step was normalized to the maximum peak inward current of each oocyte. Oocytes were injected with the following amounts of cRNA(s) encoding the indicated Na_v_‐channel: hNa_v_1.7/WT: 30 ng; hNa_v_1.7/A1632E: 30 ng; hNa_v_1.7/WT + hNa_v_1.7/A1632E: 60 ng (30 ng each). (c) Maximal relative persistent current of each cell is shown as it was used for statistical comparison. Data shown are individual values with means ± *SD*; hNa_v_1.7/WT, *N* = 14; hNa_v_1.7/WT + hNa_v_1.7/A1632E, *N* = 13; hNa_v_1.7/A1632E, *N* = 19. Difference of means: hNa_v_1.7/WT − hNa_v_1.7/WT + hNa_v_1.7/A1632E: −8.8%, 95% CI of difference of means {−13.9; −3.8}; hNa_v_1.7/WT − hNa_v_1.7/A1632E: −29.3 {−33.9; −24.7}; hNa_v_1.7/WT + hNa_v_1.7/A1632E − hNa_v_1.7/A1632E: −20.5 {−25.2; −15.7}. ^*^ *P* < 0.05, significantly different as indicated. (d) Relative persistent current of oocytes expressing the hNav1.7/A1632E channel in absence or presence of difopein. The measured persistent current at each voltage step was normalized to the maximum peak inward current of each oocyte. Oocytes were injected with the following amounts of cRNA(s) encoding either the hNav1.7/A1632E channel or the hNav1.7/A1632E channel + difopein: hNav1.7/A1632E: 30 ng; hNav1.7/A1632E + difopein: hNav1.7/A1632E 30 ng + difopein 12 ng. (e) Maximal relative persistent current of each cell is shown as it was used for statistical comparison. Data shown are individual values with means ± *SD*; hNa_v_1.7/A1632E, *N* = 19; hNa_v_1.7/A1632E + difopein, *N* = 16. Analyzed using a Mann‐Whitney test. Difference between medians: −13.6%, 95% CI of difference of means: {−18.5; −11.4}. For exact numbers of mean, *SD*, and *N* of each data point (b, d), please refer to Tables S10‐S14 ^*^ *P* < 0.05, significantly different as indicated

In agreement with the findings in HEK cells, the oocyte‐expressed hNa_v_1.7/A1632E mutant showed a prominent persistent current compared to hNa_v_1.7/WT (Figure 7a–c). As observed in HEK cells, co‐expression of the hNa_v_1.7/A1632E mutant with hNa_v_1.7/WT resulted in disproportionally reduced persistent currents, compared with the single expression of hNa_v_1.7/A1632E (Figure 7a–c, Figure S3, Supplementary Information). Adding difopein to hNa_v_1.7/A1632E in *X. laevis* oocytes reduced the persistent current of the mutant significantly (Figure 7d,e) comparable to that observed with the HEK cell expression system (Figures 1, 3, and 6).

Thus, functional dimerization of Na_v_1.7 channels seems to enhance the disease‐causing persistent current of hNa_v_1.7/A1632E, indicating that channel dimerization may be an important modifying feature for disease‐causing VGSC mutations.

## 4 DISCUSSION

In this study, we showed that (a) the pain‐linked hNa_v_1.7/A1632E mutation impairs the allosteric fast inactivation mechanism of the channel, (b) Na_v_1.7 channels form functional dimers, and (c) functionally uncoupling dimers reduces the disease‐relevant persistent current of the hNa_v_1.7/A1632E mutant channel.

### 4.1 Allosteric fast inactivation is impaired in the hNa_v_1.7/A1632E mutant, leading to persistent currents

The prominent persistent current of hNa_v_1.7/A1632E channels is likely to underlie the symptoms associated with PEPD (Eberhardt et al., 2014). Persistent currents are generally thought to be caused by impaired fast inactivation (Dib‐Hajj et al., 2013). Until very recently, fast inactivation was believed to result from the direct blockage of the channel pore by the IFM motif (Goldin, 2003; West et al., 1992), but recently published cryo‐EM channel structures suggest an allosteric mechanism for fast inactivation (Clairfeuille et al., 2019; Pan et al., 2018; Shen et al., 2017, 2019; Yan et al., 2017). According to the allosteric mechanism, the IFM motif binds to a hydrophobic pocket composed of specific residues on S5 and S6 of domains III and IV, including A1632 (Figure 2a). IFM binding induces allosteric changes of the pore‐lining S5 and S6, which close the permeation pathway. Substitution of A1632 with the larger, and better hydrated, glutamate is likely to sterically hinder the binding of the IFM motif in its pocket. This severe impairment is the likely cause of the prominent persistent current observed in hNa_v_1.7/A1632E channels.

Previous studies have replaced A1632 with aspartate, which is smaller than glutamate but also carries a negative charge, leading to even larger persistent currents (Eberhardt et al., 2014). Substitution with an uncharged amino acid such as threonine (Eberhardt et al., 2014) or glycine (Yang et al., 2016) had no effect on persistent current but shifted the voltage dependence of steady‐state fast inactivation towards more depolarized potentials, suggesting that binding is still possible, but energetically less favourable. In contrast, conservative side chain substitutions (such as A1632V) had no effect on either the persistent current or the steady‐state fast inactivation (Eberhardt et al., 2014). Thus, introduction of a negative charge into the IFM‐binding site seems to interfere with IFM binding, resulting in persistent currents. Our findings support the notion that disruption of the hydrophobic binding pocket for the IFM motif impairs fast inactivation.

Fast inactivation can be entered from the open or the closed state: While open‐state fast inactivation appears following channel activation and pore opening, the closed‐state fast inactivation does not require pore opening (Aldrich & Stevens, 1983; Bean, 1981). Both ways of fast inactivation need the VSDIV to move within the electric field, but the amount of movement has been debated in various studies: A toxin study showed that, for closed‐state inactivation, VSDIV is likely to reside in an intermediate position, which is sufficient for the IFM motif to bind while the channel remains closed (Groome, Lehmann‐Horn, & Holzherr, 2011). Open‐state fast inactivation, on the other hand, most likely needs the VSDIV to be fully activated (Armstrong, 2006; Bähring & Covarrubias, 2011; Cha, Ruben, George, Fujimoto, & Bezanilla, 1999; Groome et al., 2011).

A recent structural study using toxins to fix VSDIV of Na_v_1.7 channels in the deactivated state gave some important insights into how the movement of this channel domain and thus fast inactivation may occur (Clairfeuille et al., 2019). Comparison with VSDIV of Na_v_1.4 channels in the activated position (Pan et al., 2018) suggested two switches, that stabilize the deactivated, non‐inactivated position: Switch 1 formed an electrostatic bridge between the R5, R8 gating charges together with K6, K7 on the S4‐S5 linker and negative charges on the α1‐helix of the C‐terminus; and switch 2 formed by an acidic residue on DIV S6 and positive charges on the helix of DIII ‐ DIV linker, close to the IFM motif. The latter seems to hold the IFM and prevent its binding. In the course of the activation–inactivation sequence, the two switches open up to release the IFM motif to bind to its pocket (Clairfeuille et al., 2019). Thus, it seems plausible that open‐state fast inactivation occurs following release of both switches, whereas for closed‐state inactivation unbinding of only switch 2 is sufficient. The latter could be induced by the VSDIV moving into an intermediate position, and as the pore is already closed, this may suffice for IFM binding. Thus, although both, closed‐ and open‐state fast inactivation may need more or less VSDIV movement, both finally result in IFM binding in its binding pocket and inducing the allosteric mechanism to close the pore.

Traditionally, two separate gates for activation and fast inactivation have been assumed (Armstrong, 2006). The allosteric mechanism of fast inactivation could potentially explain both gating modes with the same gate: Activation and fast inactivation may occur as two states of the same gate with DIV S6 moving away from the permeation pathway during activation and being pushed towards it during fast inactivation by the IFM motif.

### 4.2 Na_v_1.7 channels form functional dimers

Our data suggest that similar to hNa_v_1.5 (Clatot et al., 2017), hNa_v_1.7 channels form functional dimers. For channel dimerization, two binding sites for 14‐3‐3 were reported: The first resides between amino acids 417 and 467 and the second between amino acids 517 and 555 on the DI ‐ DII linker (Figure S1). A 14‐3‐3‐independent channel interaction site, the so‐called α–α subunit interaction site, was reported to reside between these two 14‐3‐3‐binding sites (Clatot et al., 2017). As alignment of the suggested dimerization sites for hNa_v_1.5 channels revealed conserved amino acids in all subtypes except Na_v_1.4 channels (Figure S1), it is to be assumed that channel dimerization may be considered a general feature of VGSCs (Clatot et al., 2017).

### 4.3 Dimerization affects the extent of pain‐linked mutation‐induced hNa_v_1.7 channel gating changes

Inhibiting functional coupling of dimers by difopein co‐transfection drastically reduced the size of the persistent current of hNa_v_1.7/A1632E in two independent sets of experiments (HEK cells and *X. laevis* oocytes; Figures 6 and 7), indicating that the functional dimerization status could potentially regulate the severity of mutation‐induced gating changes. Dimerization of the hNa_v_1.7/A1632E mutant may support the symptoms of the patient, as homodimers have a large persistent current (Figure S4 Supplementary Information). Homodimers have a chance of about 25% to form if both alleles are read to the same extent in the nociceptor and can thus substantially contribute to the pathological persistent current. The A1632E dimer proportion could be regulated by binding and unbinding of 14‐3‐3 protein, thereby potentially triggering or supporting the reported pain attacks.

To date, the exact mechanism underlying the regulation of the persistent current induced by VGSC dimerization is still a matter of speculation. As the inhibitory protein 14‐3‐3 is thought to bind to the intracellular linker close to DI S6 (Clatot et al., 2017), it may slightly modify this segment's position. In the pore module DI S6 is situated close to DIV S6 (Shen et al., 2019), which may, in turn, be hampered by this proximity to form the binding pocket for the inactivation particle. Dimerization between subunits of one VGSC type may open a new field for channel regulation, which could potentially be even expanded, if the channels would form dimers between different subtypes, such as, for example, Na_v_1.7 and Na_v_1.8 channels. So far, there is no evidence for such heterodimerisation and this possibility needs to be investigated in further studies.

As gain‐of‐function mutations in hNa_v_1.7 channels lead to symptoms in heterozygous carriers (Fertleman et al., 2006; Lampert et al., 2010; Meents et al., 2019; Tang, Chen, Tang, & Jiang, 2015), new questions arise on how dimerization patterns of homo‐ and/or heterodimerization of VGSCs may influence the manifestation and progression of pain syndromes more generally. VGSC dimerization patterns could be altered by modulating the availability and binding of 14‐3‐3 proteins, possibly by varying site‐specific phosphorylation or other post‐translational modifications, or by heterodimer formation between different 14‐3‐3 isoforms (Pennington, Chan, Torres, & Andersen, 2018). Expression of 14‐3‐3 has been reported in dorsal root ganglia growth cones, suggesting that this protein may also regulate VGSC dimerization in nociceptive nerve endings (Kent et al., 2010).

In the case of hNa_v_1.7/R896Q, the absence of a sodium current is explained by deficient channel trafficking (Cox et al., 2010). Although some mutant channels do reach the cell membrane, these are not enough to allow a sodium current. Interestingly, for hNa_v_1.5 channels, the co‐expression of a trafficking‐deficient mutant with WT decreased total channel expression on the cell membrane (Wang et al., 2015). These reported findings may be relevant to our findings with the hNa_v_1.7/R896Q mutant. Co‐expression and dimerization of mutant and WT proteins could lead to the retention of functioning channels within the cell. This could explain why the sodium current increases in the presence of difopein, despite the concentration of transfected cDNA remaining stable. The absence of dimerization could enable the WT channel to travel to the cell membrane, thus increasing the sodium current.

We showed that although the current density was significantly reduced in the presence of both WT and hNa_v_1.7/R896Q proteins, a small sodium current remained. Our results are consistent with a recent study on Na_v_1.7 channels in induced pluripotent stem cell‐derived nociceptors from CIP patients: The authors showed that in neurons with biallelic expression of a CIP mutation, restoring one deficient Na_v_1.7 allele was sufficient to regain some, but not all, of the electrophysiological functions of these neurons (McDermott et al., 2019). Channel dimerization may reduce the current density, but the remaining level of channel function seems still sufficient for pain perception. This is supported by the fact that heterozygous parents of CIP patients do not report obvious lack of pain even though they carry a mutated allele themselves (Cox et al., 2010). Furthermore, recent studies on antagonists of Na_v_1.7 channels suggested that sodium current needs to be reduced by 80–90% (Mulcahy et al., 2019) in order to successfully achieve analgesia. Here, we report a reduction to approximately 31%, which is likely to be sufficient for action potential generation and, thus, normal pain perception. Our report of the current reduction due to dimerization between hNa_v_1.7/R896Q and hNa_v_1.7/WT is hence consistent with the prevailing knowledge on CIP and analgesia.

Thus, it appears that only one Na_v_1.7 allele is needed to produce enough Na_v_1.7 channel current to support pain perception, and therefore, only homozygous or compound heterozygous CIP mutations occur in patients. In contrast to hNa_v_1.7/R896Q, the hNa_v_1.7/G375Afs truncation mutant does not seem to dimerize with the WT protein, probably because the mutant channel is truncated before the presumed dimerization site (Clatot et al., 2017; Shorer et al., 2014). It is also possible that inadequate expression prevented dimerization. However, as the expression levels of the other mutants were sufficient and 14‐3‐3 protein is abundant in our expression system (Figure S2), we would argue that the conditions were suitable for dimerization and that the absence of the putative dimerization site is the more likely reason for the lack of effect of difopein.

Pain syndromes are complex diseases: Different phenotypes can appear in different members of a single family bearing the same mutation (Michiels, te Morsche, Jansen, & Drenth, 2005), and the exact relationship between electrophysiology and phenotype remains uncertain (Emery et al., 2015; Hampl et al., 2016). Channel dimerization is a new aspect to consider when explaining pain syndromes and their complex phenotypes. Additionally, diseases caused by different VGSC subtypes might also need to be re‐evaluated in the light of possible channel dimerization.

In conclusion, our results have shown that hNa_v_1.7 channels form functional dimers, that dimerization modifies the phenotype of the pain‐linked hNa_v_1.7/A1632E substitution mutant, and that the modified phenotype is most likely a consequence of the allosteric inactivation mechanism suggested by recently published VGSC structures. Our work supports the concept of sodium channel dimerization and its physiological function and demonstrates its relevance to human pain syndromes.

## AUTHOR CONTRIBUTIONS

A.H.R. participated in study design, performed and analysed PCR, patch‐clamp experiments, discussed and interpreted the results of the study, and wrote the manuscript. J.K. performed protein structural analysis and discussed and interpreted the results of the study. R.H. performed and analysed TEVC experiments and discussed and interpreted the results of the study. N.B. performed the biochemical experiments. C.N. performed and analysed TEVC experiments. S.D. subcloned the vectors for oocyte expression and performed the biochemical experiments. P.H. performed the mutagenesis experiments and performed and analysed the PCR experiments. C.A.B. performed and analysed molecular dynamics simulations. J.M. designed and interpreted the patch‐clamp experiments and discussed and interpreted the results of the study. J.P.M. advised on the set‐up and analysis of molecular dynamics simulations and discussed and interpreted the results of the study. G.S. designed the biochemical experiments, analysed and interpreted the results, and discussed and interpreted the results of the study. A.L. conceived and designed the study and discussed and interpreted the results of the study. All authors agree to be accountable for all aspects of the work in ensuring that questions related to the accuracy or integrity of any part of the work are appropriately investigated and resolved. All persons designated as authors qualify for authorship, and all those who qualify for authorship are listed.

## CONFLICT OF INTEREST

The authors declare no conflict of interest.

## DECLARATION OF TRANSPARENCY AND SCIENTIFIC RIGOUR

This Declaration acknowledges that this paper adheres to the principles for transparent reporting and scientific rigour of preclinical research as stated in the BJP guidelines for Design & Analysis and Animal Experimentation, and as recommended by funding agencies, publishers and other organisations engaged with supporting research.

## Supporting information


**Figure S1.** Alignment of the hNav1.5 and hNav1.7 dimerization sites. (a) Schematic diagram of hNav1.5/1.7, showing the suggested 14‐3‐3 interaction sites that support dimerization. (b) Tandem alignment of the amino acid sequences of the 3′ end of the S6 segment of domain I and the beginning of the DI–DIII linker of Nav1.5 with each of the known Navs. Conserved amino acids are shaded in grey and similar amino acids are marked by “+”. Serines conserved between Nav1.5 and Nav1.7 are highlighted in yellow—these residues may act as interaction sites for 14‐3‐3
**Figure S2**. Expression of 14‐3‐3 isoforms in HEK cells and *Xenopus laevis* oocytes. (a) PCR analysis of 14‐3‐3 isoform mRNA expression in the Nav1.7 stable cell line (lane A) and untransfected HEK293T cells (lane B). Lane C, negative control (water). (b) PCR analysis of 14‐3‐3 isoform mRNA expression in X. laevis (lane X). Lane C, negative control (water)
**Figure S3.** Persistent current of hNav1.7/WT, hNav1.7/A1632E, and its combination expressed in *Xenopus laevis* oocytes. Maximal relative persistent was used for statistical analysis. The maximal persistent current of each cell is shown in this panel. Maximal persistent current: hNav1.7/WT: 6.6% ± 2.2%, N = 14; hNav1.7/WT + hNav1.7/A1632E: 15.5% ± 5.8%, N = 13, hNav1.7/A1632E: 35.9% ± 6.3%, N = 19; hNav1.7/WT + difopein: 5.3% ± 2.6%, N = 17; hNav1.7/WT + hNav1.7/A1632E + difopein: 9.0% ± 2.1%, N = 8, hNav1.7/A1632E + difopein: 20.4% ± 2.6%, N = 16. *P < 0.05. Difference of means: hNav1.7/WT − hNav1.7/WT + hNav1.7/A1632E: −8.8% {−13.8; −3.9}; hNav1.7/WT − hNav1.7/A1632E: −29.3% {−33.8; −24.7}; hNav1.7/WT − hNav1.7/A1632E + difopein: −13.8% {−18.5; −9.1}; hNav1.7/WT + hNav1.7/A1632E − hNav1.7/A1632E: −20.5 {−25.1; −15.8}; hNav1.7/WT + hNav1.7/A1632E − hNav1.7/WT + difopein: 10.1% {5.5; 14.8}; hNav1.7WT + hNav1.7/A1632E − hNav1.7/WT + hNav1.7/A1632E + difopein: −6.5% {0.7; 12.3}; hNav1.7/WT + hNav1.7/A1632E − hNav1.7/A1632E + difopein: −4.9% {−9.8; −0.1}; hNav1.7/A1632E − hNav1.7/WT + difopein: 30.6% {26.4; 34.8}; hNav1.7/A1632E − hNav1.7/WT + hNav1.7/A1632E + difopein: 26.9% {21.5; 32.3}; hNav1.7/A1632E − hNav1.7/A1632E + difopein: 15.5% {11.1; 19.9}; hNav1.7/WT + difopein − hNav1.7/A1632E + difopein: −15.1 {−19.5; −10.7}; hNav1.7/WT + hNav1.7/A1632E + difopein − hNav1.7/A1632E + difopein: −11.4% {−17; −5.8}. Data are shown as the mean ± SD
**Figure S4**. Transfection of hNav1.7/A1632E into the Nav1.7 stable cell line in the presence of difopein. Maximal relative persistent was used for statistical analysis. The maximal persistent current of each cell is shown in this panel. Maximal persistent current: hNav1.7/WT: 2.0% ± 1.2%, N = 28; hNav1.7/WT + hNav1.7/A1632E: 4.9% ± 4.3%, N = 35; hNav1.7/A1632E: 21.8% ± 6.0%, N = 17; hNav1.7/WT + difopein: 2.2% ± 1.3, N = 26; hNav1.7/WT + hNav1.7/A1632E + difopein: 2.6% ± 1.2%, N = 24; hNav1.7/A1632E + difopein: 8.6% ± 4.0%, N = 13. *P < 0.05. Difference of means: hNav1.7/WT − hNav1.7/WT + hNav1.7/A1632E: −2.9%, 95% CI of means: {−5.4; −0.4}; hNav1.7/WT − hNav1.7/A1632E: −19.8% {−22.9; −16.8}; hNav1.7/WT − hNav1.7/A1632E + difopein: −6.6% {−9.9; −3.3}; hNav1.7/WT + hNav1.7/A16323 − hNav1.7/A1632E: −16.9% {−19.7; −14.0}; hNav1.7/WT + hNav1.7/A1632E − hNav1.7/WT + difopein: 2.7% {0.1; 5.3}; hNav1.7/WT + hNav1.7/A1632E − hNav1.7/A1632E + difopein: −3.7% {−6.9; −0.5}; hNav1.7/A1632E − hNav1.7/WT + difopein: 19.6% {16.5; 22.7}; hNav1.7/A1632E − hNav1.7/WT + hNav1.7/A1632E + difopein: 19.2% {16.1; 22.3}; hNav1.7/A1632E − hNav1.7/A1632E + difopein: 13.2% {9.6; 16.9}; hNav1.7/WT + difopein − hNav1.7/A1632E + difopein: −6.4% {−9.8; −3.0}; hNav1.7/WT + hNav1.7/A1632E + difopein − hNav1.7/A1632E + difopein: −6.0% {−9.4; −2.5}. Concentration of transfected cDNA: WT ± difopein: Nav1.7 stable cell line ± difopein; WT + A1632E ± difopein: 1.25 μg hNav1.7/A1632E + 0.25 μg GFP or difopein (Nav1.7 stable cell line); A1632E ± difopein: 1.25 μg hNav1.7/A1632E + 0.25 μg GFP or difopein (HEK293T cells). Data are shown as the mean ± SD
**Figure S5**. Current density and persistent current of cells expressing either WT, mutant, or a combination of both in the Nav1.7 stable cell line. Maximal relative persistent was used for statistical analysis. The maximal persistent current of each cell is shown in this panel. Maximal persistent current: hNav1.7/WT: 2.0% ± 1.2%, N = 28; hNav1.7/WT + hNav1.7/A1632E: 4.9% ± 4.3%, N = 35; hNav1.7/A1632E: 21.8% ± 6.0%, N = 17. *P < 0.05. For difference of means and CI of difference of means, refer to Figure 3. Peak current density of each cell in pA/pF was used for statistical analysis: hNav1.7/WT: 230.6 ± 113.1, N = 28; hNav1.7/WT + hNav1.7/A1632E: 287.4 ± 231.6, N = 35; hNav1.7/A1632E: 228.2 ± 187.6, N = 17. P > 0.05. Difference of means: hNav1.7/WT − hNav1.7/WT + hNav1.7/A1632E: −56.7, 95% CI of difference of means: {−173.6; 60.2}; hNav1.7/WT − hNav1.7/A1632E: 2.4 {−139.4; 144.2}; hNav1.7/WT + hNav1.7/A1632E − hNav1.7/A1632E: 59.1 {−77.2; 195.4}. P values > 0.05. Concentration of transfected cDNA: WT:Nav1.7 stable cell line; WT + A1632E: 1.25 μg hNav1.7/A1632E + 0.25 μg GFP (Nav1.7 stable cell line); A1632E: 1.25 μg hNav1.7/A1632E + 0.25 μg GFP (HEK293T cells). Data are shown as the mean ± SD
**Table S1**. Forward and reverse primers used in the PCR in HEK cells for each 14‐3‐3 isoform
**Table S2**. Forward and reverse primers used in the PCR in Xenopus laevis oocytes for each 14‐3‐3 isoform
**Table S3**: Mean, SD, and N for each data point in Figure 1b—WT
**Table S4**: Mean, SD, and N for each data point in Figure 1b—A1632E
**Table S5**: Mean, SD, and N for each data point in Figure 3a—WT
**Table S6**: Mean, SD, and N for each data point in Figure 3a—WT + A1632E
**Table S7**: Mean, SD, and N for each data point in Figure 3a—A1632E
**Table S8**: Mean, SEM, and N for each data point in Figure 6a—A1632E
**Table S9**: Mean, SD, and N for each data point in Figure 6a—A1632E + Difopein
**Table S10**: Mean, SD, and N for each data point in Figure 7b—WT
**Table S11**: Mean, SD, and N for each data point in Figure 7b—WT + A1632E
**Table S12**: Mean, SD, and N for each data point in Figure 7b—A1632E
**Table S13**: Mean, SD, and N for each data point in Figure 7d—A1632E + Difopein
**Table S14**: Mean, SD, and N for each data point in Figure 7d—A1632E
