# Supplementary material for: Uncoupling sodium channel dimers restores the phenotype of a pain‐linked Nav1.7 channel mutation
Source: Br J Pharmacol. 2020 Aug 24;177(19):4481–96. doi: 10.1111/bph.15196 (PMC7484505; doi:10.1111/bph.15196)
Supplement: Supplementary file 1 — Figure S1. Alignment of the hNav1.5 and hNav1.7 dimerization sites. (a) Schematic diagram of hNav1.5/1.7, showing the suggested 14‐3‐3 interaction sites that support dimerization. (b) Tandem alignment of the amino acid sequences of the 3′ end of the S6 segment of domain I and the beginning of the DI–DIII linker of Nav1.5 with each of the known Navs. Conserved amino acids are shaded in grey and similar amino acids are marked by “+”. Serines conserved between Nav1.5 and Nav1.7 are highlighted in yellow—these residues may act as interaction sites for 14‐3‐3 Figure S2. Expression of 14‐3‐3 isoforms in HEK cells and Xenopus laevis oocytes. (a) PCR analysis of 14‐3‐3 isoform mRNA expression in the Nav1.7 stable cell line (lane A) and untransfected HEK293T cells (lane B). Lane C, negative control (water). (b) PCR analysis of 14‐3‐3 isoform mRNA expression in X. laevis (lane X). Lane C, negative control (water) Figure S3. Persistent current of hNav1.7/WT, hNav1.7/A1632E, and its combination expressed in Xenopus laevis oocytes. Maximal relative persistent was used for statistical analysis. The maximal persistent current of each cell is shown in this panel. Maximal persistent current: hNav1.7/WT: 6.6% ± 2.2%, N = 14; hNav1.7/WT + hNav1.7/A1632E: 15.5% ± 5.8%, N = 13, hNav1.7/A1632E: 35.9% ± 6.3%, N = 19; hNav1.7/WT + difopein: 5.3% ± 2.6%, N = 17; hNav1.7/WT + hNav1.7/A1632E + difopein: 9.0% ± 2.1%, N = 8, hNav1.7/A1632E + difopein: 20.4% ± 2.6%, N = 16. *P < 0.05. Difference of means: hNav1.7/WT − hNav1.7/WT + hNav1.7/A1632E: −8.8% {−13.8; −3.9}; hNav1.7/WT − hNav1.7/A1632E: −29.3% {−33.8; −24.7}; hNav1.7/WT − hNav1.7/A1632E + difopein: −13.8% {−18.5; −9.1}; hNav1.7/WT + hNav1.7/A1632E − hNav1.7/A1632E: −20.5 {−25.1; −15.8}; hNav1.7/WT + hNav1.7/A1632E − hNav1.7/WT + difopein: 10.1% {5.5; 14.8}; hNav1.7WT + hNav1.7/A1632E − hNav1.7/WT + hNav1.7/A1632E + difopein: −6.5% {0.7; 12.3}; hNav1.7/WT + hNav1.7/A1632E − hNav1.7/A1632E + difopein: −4.9% {−9.8; −0.1}; hNav1.7/A1632E − [file BPH-177-4481-s001.pdf]

Supplementary Information to

Uncoupling sodium channel dimers rescues the phenotype of a pain-linked Nav1.7 mutation

Running title: Pain-linked Nav1.7 inactivation and dimerization

Annika H. Rühlmann[1], Jannis Körner[1,2], Ralf Hausmann[3], Nikolay Bebrivenski[3], Christian Neuhof[3], Silvia Detro-Dassen[3], Petra Hautvast[1], Carène A. Benasolo[3,4], Jannis Meents[1], Jan-Philipp Machtens[3,4], Günther Schmalzing[3], and Angelika Lampert[1]

[1]Institute of Physiology, RWTH Aachen University, Pauwelsstrasse 30, 52074 Aachen, Germany
[2]Department of Anaesthesiology, RWTH Aachen University, Medical Faculty, Pauwelsstrasse 30, 52074 Aachen, Germany
[3]Institute of Clinical Pharmacology, RWTH Aachen University, Pauwelsstrasse 30, 52074 Aachen, Germany
[4]Institute of Biological Information Processing (IBI-1), Molekular- und Zellphysiologie, and JARA-HPC, Forschungszentrum Jülich, Jülich, Germany

Corresponding author:
Prof. Dr. Angelika Lampert
Institute of Physiology
RWTH Aachen University

Pauwelsstrasse 30
52074 Aachen
Germany
Tel.: +43 2041 80 88811
Email: alampert@ukaachen.de

A

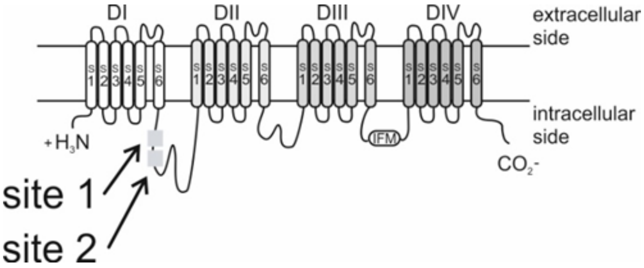

B

```
Nav1.1
14-3-3 interaction site 1:
hNav1.5 417-467  EEQNQATIAETEEKEKRFQEAMEMLKKEHEALTIRGVDTVSRSSLEMSPLA
hNav1.1 427-477  EEQNQATLEEAEQKEAEFQQMIEQLKKQQEAAQQAATATASEHSREPSAAG
                        +    +      + +     +
14-3-3 interaction site 2:
hNav1.5 517-555 RTSM--KPRSSRGSIFTFRRR-–DLGSEADFADDENSTAGESE
hNav1.1 560-602 RGSLFSPRRNSRTSLFSFRGRAKDVGSENDFADDEHSTFEDNE
                   +     +    + +       +          +    ++
Nav1.2
14-3-3 interaction site 1:
hNav1.5 417-467 EEQNQATIAETEEKEKRFQEAMEMLKKEHEALTIR---------------GVDTVSRSSLEMSPLA
hNav1.2 429-494 EEQNQATLEEAEQKEAEFQQMLEQLKKQQEEAQAAAAAASAESRDFSGAGGIGVFSESSSVASKLS
                       +    +      + +     +                       +             +
14-3-3 interaction site 2:
hNav1.5 517-555 RTSMKPRSSRGSIFTFRRR-–DLGSEADFADDENSTAGESE
hNav1.2 465-605 SLFSPRRNSRASLFSFRGRAKDIGSENDFADDEHSTFEDND
                       +    + +       +          +    +++
Nav1.3
14-3-3 interaction site 1:
hNav1.5 417-467 EEQNQATIAETEEKEKRFQEAMEMLKKEHEALT--------------IRGVDTVSRSSLEMSPLA
hNav1.3 428-492 EEQNQATLEEAEQKEAEFQQMLEQLKKQQEEAQAVAAASAASRDFSGIGGLGELLESSSEASKLS
                       +    +      + +     +                      +  +          +
14-3-3 interaction site 2:
hNav1.5 517-555 RTSMKP-------------------RSSRGSIFTFRRR—-DLGSEADFADDENSTAGESE
hNav1.3 546-605 LTSDKKFCSPHQSLLSIRGSLFSPRRNSKTSIFSFRGRAKDVGSENDFADDEHSTFEDSE
                                          + +    +       +          +    +
Alignment not possible for Nav1.4
Nav1.6
14-3-3 interaction site 1:
hNav1.5 417-467 EEQNQATIAETEEKEKRFQEAMEMLKKEHEALTIRGV----DTVSRSSLEM-------SP-----------LA
hNav1.6 415-488 EEQNQATLEEAEQKEAEFKAMLEQLKKQQEEAQAAAMATSAGTVSEDAIEEEGEEGGGSPRSSSEISKLSSKSA
                       +    +     +  +     +        +          ++
14-3-3 interaction site 2:
hNav1.5 517-555 RTSMKPRSSRGSIFTFRR----RDLGSEADFADDENSTAGESE
hNav1.6 553-595 SPFLSRHNSKSSIFSFRGPGRFRDPGSENEFADDEHSTVEESE
                   +   + +    +              +     +
Nav1.7
14-3-3 interaction site 1:
hNav1.5 417-467EEQNQATIAETEEKEKRFQEAMEMLKKEHEALTIRGVDTVSRSSLEMSPL-----------------A
hNav1.7 406-473EEQNQANIEEAKQKELEFQQMLDRLKKEQEEAEAIAAAAAEYTSIRRSRIMGLSESSSETSKLSSKSA
                          ++      + ++                   + +    +


14-3-3 interaction site 2:
hNav1.5 517-555 RTSMKPRSSRGSIFTFRR-------------RDLGSEADFADDENSTAGESE
hNav1.7 531-582 TPNQSPLSIRGSLFSARRSSRTSLFSFKGRGRDIGSETEFADDEHSIFGDNE
                  +         + +                  +    +     +    ++


Nav1.8
14-3-3 interaction site 1:
```

```
hNav1.5 417-467 EEQNQATIAETEEKEKRFQEAMEMLKKEHEALTIRGVDTVSRSSLEMSPLA
hNav1.8 401-451 EEQNQATTDEIEAKEKKFQEALEMLRKEQEVLAALGIDTTSLHSHNGSPLT
                                +    +   +          +

14-3-3 interaction site 2:
hNav1.5 517-555 RTSMKPRSSRGSIFTFRR-–RDLGSEADFADDENSTAGESE
hNav1.8 496-531 ----KRRASHGSVFHFRSPGRDISLPEGVTDD-GVFPGDHE
                       +    +         +               +


Nav1.9
14-3-3 interaction site 1:
hNav1.5 417-467 EEQNQATIAETEEKEKRFQEAMEMLKKEHEALTIRGVDTVSRSSLEMSPLA
hNav1.9 404-554 EEQNKNVAAEIEAKEKMFQEAQQLLKEEKEALVAMGIDRSSLTSLETSYFT
                    +                 ++  +         +     +
No results for the second binding site
```

Fig. S1. Alignment of the hNav1.5 and hNav1.7 dimerization sites.
(A) Schematic diagram of hNav1.5/1.7, showing the suggested 14-3-3 interaction sites that support dimerization.
(B) Tandem alignment of the amino acid sequences of the 3′ end of the S6 segment of domain I and the beginning of the DI–DIII linker of Nav1.5 with each of the known Navs. Conserved amino acids are shaded in gray and similar amino acids are marked by “+”. Serines conserved between Nav1.5 and Nav1.7 are highlighted in yellow – these residues may act as interaction sites for 14-3-3.

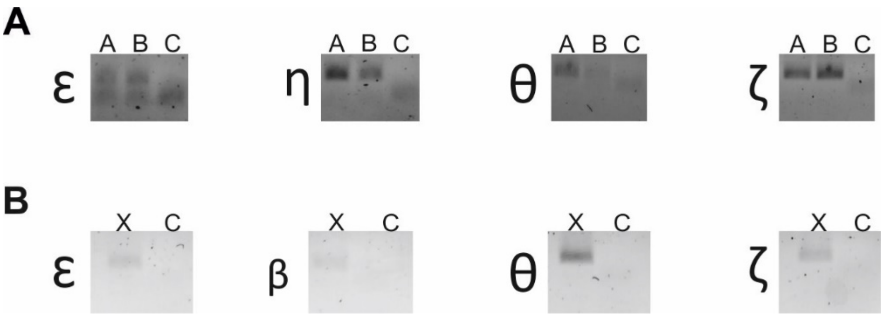

Fig. S2. Expression of 14-3-3 isoforms in HEK cells and *X. leavis* oocytes
(A) PCR analysis of 14-3-3 isoform mRNA expression in the Nav1.7 stable cell line (lane A) and untransfected HEK293T cells (lane B). Lane C, negative control (water).
(B) PCR analysis of 14-3-3 isoform mRNA expression in *X. laevis* (lane X). Lane C, negative control (water).

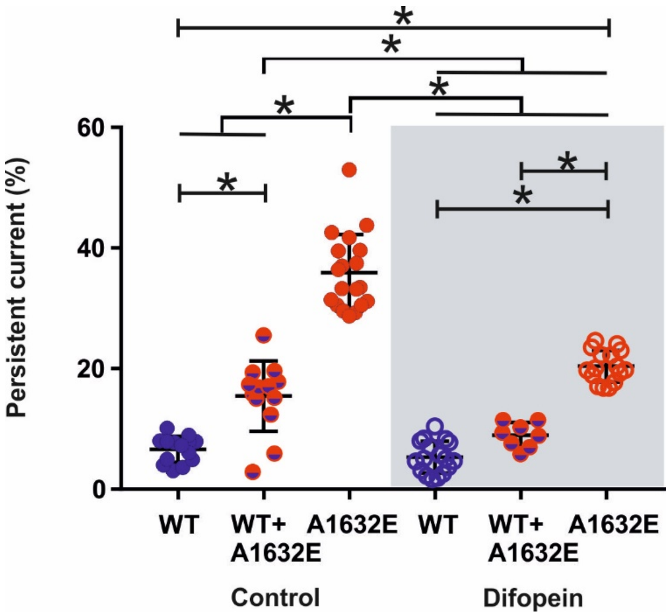

Fig. S3. Persistent current of hNav1.7/WT, hNav1.7/A1632E and its combination expressed in *X. laevis* oocytes.

Maximal relative persistent current of each cell as used for statistical comparison. Data shown are individual values with means ± *SD*: hNav1.7/WT, *N* = 14; hNav1.7/WT+hNav1.7/A1632E, *N* = 13; hNav1.7/A1632E, *N* =19; hNav1.7/WT+difopein, *N* = 17; hNav1.7/WT+hNav1.7/A1632E+difopein, *N* = 8, hNav1.7/A1632E+difopein, *N* = 16. *$p<0.05$.

Difference of means: hNav1.7/WT – hNav1.7/WT+hNav1.7/A1632E: -8.8% {-13.8;-3.9}; hNav1.7/WT – hNav1.7/A1632E: -29.3% {-33.8;-24.8}; hNav1.7/WT – hNav1.7/A1632E+difopein: -13.8% {-18.4;-9.1}; hNav1.7/WT+hNav1.7/A1632E – hNav1.7/A1632E: -20.5 {-25.0;-15.9}; hNav1.7/WT+hNav1.7/A1632E – hNav1.7/WT+difopein: 10.1% {5.5;14.8}; hNav1.7WT+hNav1.7/A1632E – hNav1.7/WT+hNav1.7/A1632E+difopein: -6.5% {0.8;12.2}; hNav1.7/WT+hNav1.7/A1632E – hNav1.7/A1632E+difopein: -4.9% {-9.7;-0.2}; hNav1.7/A1632E – hNav1.7/WT+difopein: 30.6% {26.4;34.8}; hNav1.7/A1632E – hNav1.7/WT+hNav1.7/A1632E+difopein: 26.9% {21.6;32.3}; hNav1.7/A1632E – hNav1.7/A1632E+difopein: 15.5% {11.2;19.8}; hNav1.7/WT+difopein – hNav1.7/A1632E+difopein: -15.1 {-19.5;-10.7}; hNav1.7/WT+hNav1.7/A1632E+difopein – hNav1.7/A1632E+difopein: -11.4% {-16.9;-5.9}.

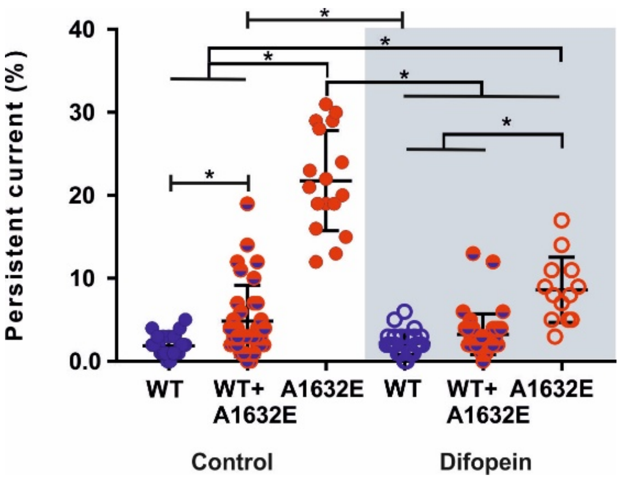

Fig. S4. Transfection of hNav1.7/A1632E into the Nav1.7 stable cell line in the presence of difopein.

Maximal relative persistent current of each cell as used for statistical comparison. Data shown are individual values with means ± *SD* hNav1.7/WT, *N* = 28; hNav1.7/WT+hNav1.7/A1632E, *N* = 35; hNav1.7/A1632E, *N* = 17; hNav1.7/WT+difopein, *N* = 26; hNav1.7/WT+hNav1.7/A1632E+difopein, *N* = 24; hNav1.7/A1632E+difopein, *N* = 13. *$p<0.05$.

Difference of means: hNav1.7/WT – hNav1.7/WT+hNav1.7/A1632E: -2.9%, 95% CI of means: {-5.4;-0.4 }; hNav1.7/WT – hNav1.7/A1632E: -19.8% {-22.9;-16.8}; hNav1.7/WT – hNav1.7/A1632E+difopein: -6.6% {-9.9;-3.3}; hNav1.7/WT+hNav1.7/A1632 – hNav1.7/A1632E: -16.9% {-19.9;-14.0}; hNav1.7/WT+hNav1.7/A1632E – hNav1.7/WT+difopein: 2.7% {0.1;5.3}; hNav1.7/WT+hNav1.7/A1632E – hNav1.7/A1632E+difopein: -3.7% {-6.9;-0.5}; hNav1.7/A1632E – hNav1.7/WT+difopein: 19.6% {16.5;22.7}; hNav1.7/A1632E – hNav1.7/WT+ hNav1.7/A1632E+difopein: 19.2% {16.1;22.3}; hNav1.7/A1632E – hNav1.7/A1632E+difopein: 13.2% {9.6;16.9}; hNav1.7/WT+difopein – hNav1.7/A1632E+difopein: -6.4% {-9.8;-3.0}; hNav1.7/WT+hNav1.7/A1632E+difopein – hNav1.7/A1632E+difopein: -6.0% {-9.4;-2.5}.

Concentration of transfected cDNA: WT ± difopein: Nav1.7 stable cell line ±difopein; WT+A1632E±difopein: 1.25µg hNav1.7/A1632E + 0.25µg GFP or difopein (Nav1.7 stable cell line); A1632E ± difopein: 1.25µg hNav1.7/A1632E + 0.25µg GFP or difopein (HEK293T cells).

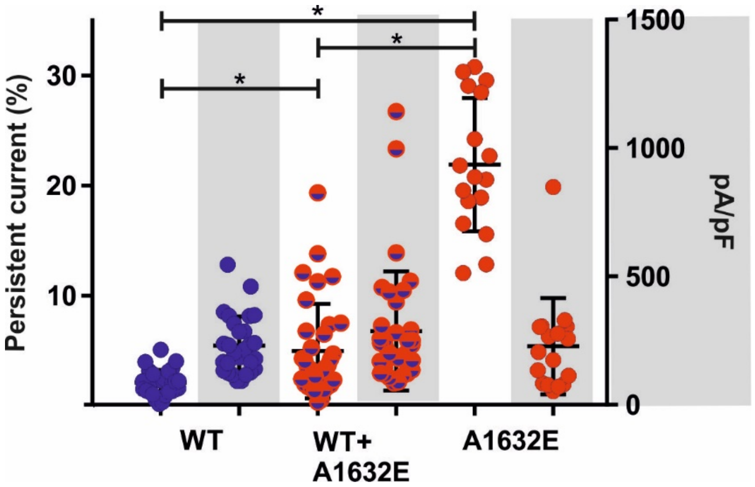

Fig. S5. Current density and persistent current of cells expressing either WT, mutant or a combination of both in the Nav1.7 stable cell line.

Maximal relative persistent current of each cell as used for statistical comparison. Data shown are individual values with means ± *SD* hNav1.7/WT, N = 28; hNav1.7/WT+hNav1.7/A1632E, N = 35; hNav1.7/A1632E, *N* = 17. *p<0.05. For difference of means and CI of difference of means, refer to Fig. 3.

Peak current density of each cell in pA/pF was used for statistical analysis: hNav1.7/WT: 230.6 ± 113.1, *N* = 28; hNav1.7/WT+hNav1.7/A1632E: 287.4 ± 231.6, *N* = 35; hNav1.7/A1632E: 228.2 ± 187.6, *N* = 17. P > 0.05. Difference of means: hNav1.7/WT – hNav1.7/WT+hNav1.7/A1632E: -56.7, 95% CI of difference of means: {-173.6;60.2}; hNav1.7/WT – hNav1.7/A1632E: 2.4 {-139.4;144.2 }; hNav1.7/WT+hNav1.7/A1632E – hNav1.7/A1632E: 59.1 {-77.2;195.4}.

P values > 0.05.

Concentration of transfected cDNA: WT:Nav1.7 stable cell line; WT+A1632E: 1.25µg hNav1.7/A1632E + 0.25µg GFP (Nav1.7 stable cell line); A1632E: 1.25µg hNav1.7/A1632E + 0.25µg GFP (HEK293T cells).

Table S1. Forward and reverse primers used in the PCR in HEK cells for each 14-3-3 isoform.

14-3-3 isoform	Forward primer	Reverse primer
Beta	GTG GCA TCT GGA GAC AAC AAA C	AGA CGA ATT GGG TGT GTA GGC
Epsilon	CTG AGC GAT ACG ACG AAA TGG	ATT CTC CAG GAG GCT CTT CTA G
Eta	ACG ACA TGG CCT CCG CTA	GCT CAA TGC TGC TAA TGA CCC T
Gamma	GCG AAG ATG GTG GAC CGC	GTT CCT CAT TCG ACA GTG GCT
Sigma	GGA GGG TGC TGT CCA GTA TTG	TTG ATG AGG TGG CTG TCC AG
Theta	CAC GGT GCT GGA ATT GTT GG	CGA TCA TCA CCA CAC GCA AC
Zeta	ACT ACC GTT ACT TGG CTG AGG	AGA CCC AGT CTG ATA GGA TGT G

Table S2. Forward and reverse primers used in the PCR in X.laevis oocytes for each 14-3-3 isoform.

14-3-3 isoform	Forward primer	Reverse primer
Beta	GAC TTG GCC TGG CTC TAA AC	AGT GTG TCC AGT TCA GCT ATC G
Epsilon	AGC AGG CCG AGA GAT ATG AC	ACG CTC TCC TGG CTC CAA TC
Eta	GCA GGC GGA GAG ATA TGA GG	TTA TCA CAC GCC ACG AGG AG
Gamma	TCA CCG CAA CAT GGT GGA C	GTT CTT CGT TGG AGA GTG CTT C
Theta	AGC AGA AGT CGG ACA GTG AAG	GGC TTT CCG TTG CAG TTG AAG
Zeta	TTG CCA ATG CCA CGC AAC	TTG ATA CGC CTT CTG TGA CTG

Table S3: Mean, SD and N for each data point in Fig. 1B - WT

Voltage (mV)	Mean (mV)	SD	N
-90	0,02	0,8	28
-80	-0,47	0,9	28
-70	-0,14	1,0	28
-60	-0,32	1,0	28
-50	-0,57	1,1	28
-40	-0,78	0,9	28
-30	-0,54	1,9	28
-20	-0,25	1,2	28
-10	-0,42	1,2	28
0	-0,42	1,5	28
10	-0,35	1,4	28
20	0,3	1,6	28
30	0,09	2,0	28
40	1,06	2,2	28

Table S4: Mean, SD and N for each data point in Fig.1B – A1632E

Voltage (mV)	Mean (mV)	SD	N
-90	-0,31	1,6	17
-80	-0,62	1,8	17
-70	-1,7	3,2	17
-60	-1,02	2,9	17
-50	-1,96	2,0	17
-40	-4,84	3,6	17
-30	-10,27	3,3	17
-20	-16,94	6,2	17
-10	-18,7	6,5	17
0	-20,4	6,6	17
10	-17,81	5,1	17
20	-12,18	5,8	17
30	-8,53	4,0	17
40	-2,43	3,5	17

Table S5: Mean, SD and N for each data point in Fig. 3A - WT

Voltage (mV)	Mean (mV)	SD	N
-90	0,02	0,8	28
-80	-0,47	0,9	28

-70	-0,14	1,0	28
-60	-0,32	1,1	28
-50	-0,57	1,1	28
-40	-0,78	0,9	28
-30	-0,54	1,0	28
-20	-0,25	1,2	28
-10	-0,42	1,2	28
0	-0,42	1,5	28
10	-0,35	1,4	28
20	0,3	1,6	28
30	0,09	2,0	28
40	1,06	2,2	28

Table S6: Mean, SD and N for each data point in Fig. 3A – WT + A1632E

Voltage (mV)	Mean (mV)	SD	N
-90	-0,21	1,3	35
-80	-0,11	1,2	35
-70	0,07	1,6	35
-60	-0,83	1,9	35
-50	-1,15	1,4	35
-40	-1,78	2,0	35
-30	-2,78	2,7	35
-20	-3,04	4,1	35
-10	-3,62	4,6	35
0	-3,51	4,0	35
10	-2,5	3,7	35
20	-1,96	3,3	35
30	-0,91	2,9	35
40	0,38	2,4	35

Table S7: Mean, SD and N for each data point in Fig. 3A – A1632E

Voltage (mV)	Mean (mV)	SD	N
-90	-0,31	1,6	17
-80	-0,62	1,8	17
-70	-1,7	3,2	17
-60	-1,02	2,9	17
-50	-1,96	2,0	17
-40	-4,84	3,6	17
-30	-10,27	3,4	17
-20	-16,94	6,2	17
-10	-18,7	6,5	17

0	-20,4	6,6	17
10	-17,81	5,1	17
20	-12,18	5,8	17
30	-8,53	4,0	17
40	-2,43	3,5	17

Table S8: Mean, SEM and N for each data point in Fig. 6A – A1632E

Voltage (mV)	Mean (mV)	SD	N
-90	-0,31	1,6	17
-80	-0,62	1,8	17
-70	-1,7	3,2	17
-60	-1,02	2,9	17
-50	-1,96	2,0	17
-40	-4,84	3,6	17
-30	-10,27	3,4	17
-20	-16,94	6,2	17
-10	-18,7	6,5	17
0	-20,4	6,6	17
10	-17,81	5,1	17
20	-12,18	5,8	17
30	-8,53	4,0	17
40	-2,43	3,5	17

Table S9: Mean, SD and N for each data point in Fig. 6A – A1632E+Difopein

Voltage (mV)	Mean (mV)	SD	N
-90	-1,22	2,6	13
-80	0,0	3,7	13
-70	-0,63	2,3	13
-60	-1,81	3,2	13
-50	-2,93	1,8	13
-40	-2,7	2,3	13
-30	-5,76	3,0	13
-20	-6,28	3,4	13
-10	-5,52	4,0	13
0	-4,56	2,7	13
10	-3,92	4,3	13
20	-4,62	4,5	13
30	-3,62	4,4	13
40	0,85	6,8	13

Table S10: Mean, SD and N for each data point in Fig. 7B – WT

Voltage (mV)	Mean (mV)	SD	N
-90	0,94	1,2	14
-80	1,13	1,6	14
-70	0,72	1,6	14
-60	0,46	2,2	14
-50	0,02	2,3	14
-40	-1,01	3,7	14
-30	-3,96	3,4	13
-20	-6,62	2,2	14
-10	-3,70	3,1	14
0	-1,79	4,4	13
10	1,43	3,0	13
20	1,26	5,0	13
30	-0,06	4,6	13
40	-0,19	4,8	12

Table S11: Mean, SD and N for each data point in Fig. 7B – WT + A1632E

Voltage (mV)	Mean (mV)	SD	N
-90	0,99	1,1	17
-80	0,88	1,4	17
-70	0,55	1,3	17
-60	0,55	1,6	17
-50	-0,39	1,8	17
-40	-4,21	4,2	17
-30	-10,91	3,2	13
-20	-15,44	5,8	13
-10	-12,85	4,4	13
0	-7,43	3,6	13
10	-3,19	2,8	13
20	-0,11	3,1	13
30	-0,44	3,4	12
40	-0,53	4,3	12

Table S12: Mean, SD and N for each data point in Fig. 7B – A1632E

Voltage (mV)	Mean (mV)	SD	N
-90	0,29	0,3	20
-80	0,11	0,4	20
-70	-0,19	0,6	20

-60	-0,4	1,0	20
-50	-1,53	1,1	20
-40	-8,42	2,9	18
-30	-30,63	7,7	16
-20	-35,9	6,3	19
-10	-31,03	6,6	15
0	-21,75	6,1	15
10	-15,47	5,5	16
20	-9,81	5,1	15
30	-4,77	6,0	20
40	-2,59	6,1	20

Table S13: Mean, SD and N for each data point in Fig. 7D – A1632E+Difopein

Voltage (mV)	Mean (mV)	SD	N
-90	0,05	0,8	18
-80	-0,27	0,9	18
-70	-0,15	0,7	18
-60	-0,52	1,0	18
-50	-2,07	1,2	18
-40	-9,09	2,6	18
-30	-19,65	-2,8	14
-20	-20,39	2,5	16
-10	-15,87	2,5	16
0	-10,13	3,1	18
10	-6,82	3,2	18
20	-4,36	3,7	18
30	-2,34	3,8	18
40	-0,21	4,3	18

Table S14: Mean, SD and N for each data point in Fig. 7D – A1632E

Voltage (mV)	Mean (mV)	SD	N
-90	0,29	0,3	20
-80	0,11	0,4	20
-70	-0,19	0,6	20
-60	-0,4	1,0	20
-50	-1,53	1,1	20
-40	-8,42	2,9	18
-30	-30,63	7,7	16
-20	-35,9	6,3	19
-10	-31,03	6,6	15

0	-21,75	6,1	15
10	-15,47	5,5	16
20	-9,81	5,1	15
30	-4,77	6,0	20
40	-2,59	6,1	20

1
